# Traditional Chinese Medicine as a Promising Strategy for the Treatment of Alzheimer’s Disease Complicated With Osteoporosis

**DOI:** 10.3389/fphar.2022.842101

**Published:** 2022-06-01

**Authors:** Weifan Xu, Yiping Jiang, Nani Wang, Huanhuan Bai, Shengyan Xu, Tianshuang Xia, Hailiang Xin

**Affiliations:** ^1^ Department of Pharmacognosy, School of Pharmacy, Naval Medical University, Shanghai, China; ^2^ Department of Pharmacy, Fujian University of Traditional Chinese Medicine, Fuzhou, China; ^3^ Department of Medicine, Zhejiang Academy of Traditional Chinese Medicine, Hangzhou, China

**Keywords:** Alzheimer’s disease, osteoporosis, pathophysiological link, action mechanism, traditional Chinese medicine

## Abstract

Alzheimer’s disease (AD) and osteoporosis (OP) are progressive degenerative diseases caused by multiple factors, placing a huge burden on the world. Much evidence indicates that OP is a common complication in AD patients. In addition, there is also evidence to show that patients with OP have a higher risk of AD than those without OP. This suggests that the association between the two diseases may be due to a pathophysiological link rather than one disease causing the other. Several *in vitro* and *in vivo* studies have also proved their common pathogenesis. Based on the theory of traditional Chinese medicine, some classic and specific natural Chinese medicines are widely used to effectively treat AD and OP. Current evidence also shows that these treatments can ameliorate both brain damage and bone metabolism disorder and further alleviate AD complicated with OP. These valuable therapies might provide effective and safe alternatives to major pharmacological strategies.

## Introduction

With the advent of an aging society, aging-related diseases have occupied the forefront of the disease spectrum in China. Alzheimer’s disease (AD) and osteoporosis (OP) are two common aging-related diseases in clinic, which have seriously threatened the health of middle-aged and elderly people. AD is a disease dominated by a series of central neurodegenerative conditions with hidden pathogenesis and unknown etiology (Hogh, 2017). It is clinically characterized by memory impairment, aphasia, impairment of visuospatial skills, executive dysfunction, and personality and behavior changes (Zhang and Zheng, 2019), which badly affect patients’ life. The pathologic hallmarks of AD are cerebral extracellular amyloid plaques and intracellular neurofibrillary tangles, but not all people with amyloid plaques or neurofibrillary tangles develop AD (Weller and Budson, 2018). However, the underlying pathophysiological mechanism is still elusive, especially for older adults. OP is a degenerative bone disease, which leads to a decrease in bone mineral density (BMD) and bone mass due to various reasons, and the degeneration of bone micro-structure increases bone brittleness, thus increasing the risk of fracture (Aspray and Hill, 2019). The pathogenesis of OP is considered to include the defect in bone micro-architecture, poor intrinsic material properties of bone, defective repair of micro-damage to the bone, and excessive bone remodeling (Armas and Recker, 2012). These points provide different treatment strategies for OP, as well as expanding the possibilities of new drug screening. In addition, clinical studies have found that patients diagnosed with AD are often accompanied by OP and hip fracture, and the BMD of patients with AD is often lower than that of the non-AD population (Chen and Lo, 2017), indicating that there is a correlation between the pathogenesis of AD and OP, and patients are prone to the symptoms of these two diseases.

**FIGURE 1:**
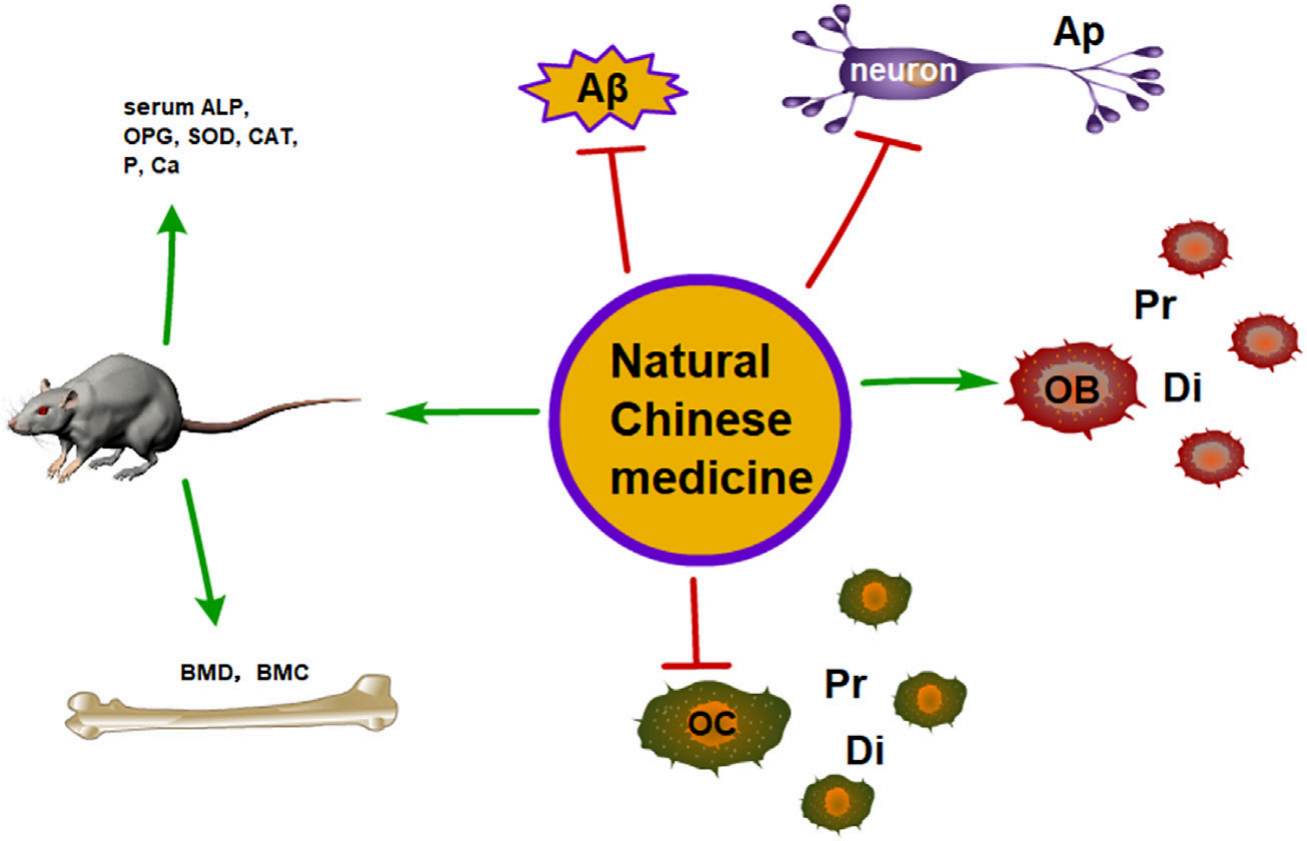
The therapeutic potential of the natural Chinese medicine for the treatment of AD and OP (Ap, apoptosis; Pr, proliferation; Di, differentiation).

Traditional Chinese medicine (TCM) has become increasingly popular thanks to its fewer side effects and high effectiveness in treating diseases. TCM formula is usually composed of more than one kind of herb, and the purpose of the herbal formula is to improve the therapeutic effects or decrease the possible side effects of a single herb through the complex interactions among herbs. Meanwhile, TCM is rich in natural components, which can provide promising ideas for the screening and research of new drugs. The TCM formula has been widely used in effectively treating a variety of aging-related diseases, including AD and OP. Some are the most classical and specific drugs, such as *Morinda officinalis* How., *Eucommia ulmoides* Oliv., *Lycium barbarum* L., *Atractylodes macrocephala* Koidz., *Salvia miltiorrhiza* Bge., *Rehmannia glutinosa* Libosch., and *Glycyrrhiza uralensis* Fisch., *Cistanche deserticola* Ma., when applied to the treatment of dementia and bone loss, with beneficial effects on the growth and development of the nervous system and skeleton tissues (Figure 1) (Howes et al., 2017; Pei et al., 2020; Zhang ND et al., 2016; Wang T et al., 2017). Therefore, we review the link between AD and OP from the epidemiological perspective and the underlying molecular mechanism and introduce some natural Chinese medicines for treating AD complicated with OP to provide a reference for future clinical treatment.

## Correlation Between Alzheimer’s Disease and Osteoporosis

### The Epidemiology of AD and OP

AD is one of the most common diseases in elderly people. The World Health Organization (WHO) estimated that there was a strong link between age and the prevalence of AD, and the global prevalence of AD in the elderly (over 60) was as high as 5%–7% (Prince et al., 2013). In addition, AD is also related to sex, and recent studies have found that nearly two-thirds of AD patients are women (Kim et al., 2018). According to the Alzheimer’s Disease International (ADI) statistics, over 55 million people suffer from AD worldwide. This staggering figure grew rapidly and was predicted to reach 78 million by 2030 (Alzheimer’s Disease International, McGill University, 2021). In addition, as the number of AD patients continues to rise, the cost of AD treatment also rises. A report shows that the total cost of global AD treatment in 2020 was 305 billion US dollars (Wong, 2020). In China, the total cost of AD treatment in 2015 was 167.74 billion US dollars, and the total cost was expected to reach 507.49 billion US dollars in 2030 (Jia et al., 2018). These numbers reveal that AD has imposed a huge burden on the world economy. More precariously, according to a recent AD report in the United States, the number of AD patients and their cost have been increasing rapidly, despite an important breakthrough made in the prevention, mitigation, and treatment of AD, especially after the outbreak of COVID-19 (Alzheimer’s Disease International, McGill University, 2021).

As for osteoporosis, it was found in a survey that the incidence of fragility fractures associated with OP in 2017 was as many as 2.7 million people in European countries (Eric, 2020), and the incidence of osteoporosis in women is generally higher than that in men, especially in postmenopausal women (Lorentzon et al., 2021), who are consistent with AD. However, although bone mass loss in women is faster, the life expectancy of women is generally longer than that of men. A cohort study in Denmark found that the remaining life expectancy was 18.2 years for men with OP at the age of 50 and 7.5 years for men at the age of 75, while for women, the figure was 26.4 and 13.5 years, respectively (Abrahamsen et al., 2015). The prevalence rate of OP in China rose from 14.94% in 2008 to 27.96% in 2012–2015, and the prevalence rate increased with age (28.09% in 25–35 years old and 34.65% in people over 50 years old) (Chen et al., 2016). According to the results of China’s OP epidemiological survey in 2018, the prevalence rate of OP in people over 50 years old was 19.2%, as well as 32.0% in people over 65 years, suggesting its widespread features in middle-aged and elderly people in China (Osteoporosis and Bone Mineral Disease Branch of Chinese Medical Association, 2019). In addition, the lack of physical exercise, low vitamin D levels, smoking, drinking, and high caffeine intake can also reduce BMD, leading to OP. According to a report, the human BMD peaks at 35 years old, then begins to decline, and accelerates significantly after menopause. Hence, to prevent OP, young women, as well as men, should quit smoking, avoid excessive drinking, exercise regularly, and take in the right amount of calcium and vitamin D (Lamichhane, 2005).

In addition, the risk of OP in AD and the risk of AD in OP are two different aspects when considering the relationship between these two situations. A prospective, observational study found that lower BMD was associated with an increased risk of increasing AD (Tan et al., 2005). Dual-energy X-ray absorptiometry of the femoral neck was measured for BMD analysis in the study and the results indicated that the lowest quartile of femoral neck BMD was associated with a twice risk of AD in women but not in men, suggesting the possibility of a protective role of cumulative estrogen exposure. Compared with the general population, postmenopausal women with severe OP were found to have higher risks of AD and other dementia (Amouzougan et al., 2017). Estrogen has the potential to influence both brain aging and bone metabolism. The prevalence of AD is slightly higher in women than men, and postmenopausal women are predisposed to develop OP (Henderson, 2006; Depypere et al., 2016). AD is also considered a risk factor for OP. A population-based cohort study indicated that individuals with AD were at a higher risk of hip fracture (Wang et al., 2014). Another meta-analysis study also found that the AD population had a decreased level of hip BMD and approximately twice risk of hip fracture compared with healthy controls (Zhao et al., 2012). The circadian rhythm is usually poorly regulated, and daytime sleepiness is common in the AD population. This situation worsens as cognitive function exacerbates (Lee et al., 2007). The condition limits outdoor physical activities, which may, in turn, decrease sunlight exposure and subsequently cause vitamin D deficiency. Lack of physical activities and vitamin D deficiency may explain the higher risk of OP in the AD population (Nanes and Kallen, 2014). The relationship between AD and OP is further illustrated by the association between BMD values measured by dual-energy X-ray absorptiometry and cerebral volume measured by magnetic resonance imaging. BMD is decreased in the earliest clinical stages of AD and is connected with brain atrophy and memory loss (Loskutova et al., 2009). What is more, BMD and hypothalamic volume were demonstrated to have a positive relationship in the early AD group after controlling for age and gender (Loskutova et al., 2010).

In light of the above epidemiological statistics, it was clear that AD and OP have similarities in high morbidity and disability, strong correlation with age and gender, and high cost for treatment. Therefore, the common pathological mechanism is of great interest as it may direct the future study design for AD and OP prevention.

### The Common Pathological Mechanism of AD and OP

**FIGURE 2:**
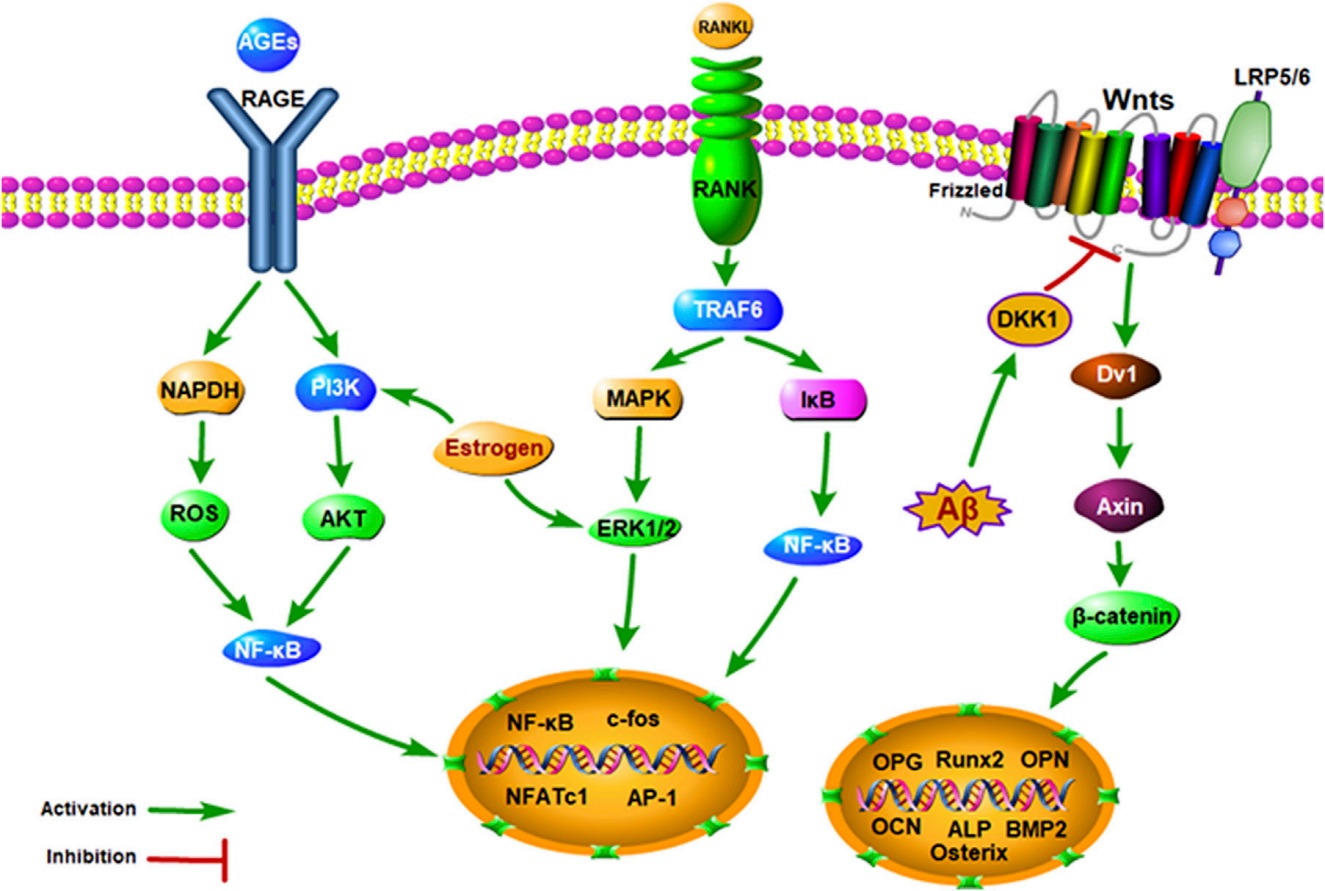
Signaling pathways involved in the common pathogenesis of AD and OP.

Modern pharmacological studies indicate that there are many common pathological mechanisms between AD and OP, such as estrogen deficiency, Wnt signaling pathway inhibition, OPG/RANK/RANKL axis disorder, NF-κB signaling pathway activation, amyloid precursor protein cleavage, vitamin D deficiency, inflammation, parathyroid hormone (PTH) deficiency, calcitonin gene-related peptide (CGRP) expression, and autonomic nervous system (ANS) dysfunction (Figure 2).

### Estrogen

Estrogen is a substance that promotes the maturation of the secondary sexual characteristics and sexual organs of the female. Estrogen receptors are distributed throughout the body, including the bones and brain. Therefore, estrogen has a wide range of important physiological effects. Estrogen can maintain the secondary sexual characteristics of women and have obvious effects on the growth and maturation of bones and the development of the brain (Patel et al., 2018). A cross-sectional study examined the BMD in women with varying degrees of cognitive impairment. It was found that cognitive impairment was related to low BMD, and cognitive impairment in postmenopausal women was significantly related to the decrease in BMD (Lee et al., 2012). This finding suggested that cognitive aging was multifactorial, and estrogen deficiency might be one of the crucial causes of cognitive impairment. By measuring the trabecular and cortical BMD of elderly women, Alice et al. found that reduced BMD showed a higher risk of cognitive decline (Laudisio et al., 2016). Similar to ovariectomized (OVX) rodents, it was found that low estrogen levels could also accelerate bone mass loss and lead to memory impairment (Eiken et al., 2014). In addition, estrogen therapy with continuous dependence can improve BMD and prevent osteoporotic fractures. Estrogen has a bone protective effect, which can promote bone formation by increasing osteoblasts (OB) and inhibiting high bone turnover so as to prevent bone loss (Stevenson, 2006). β-Amyloid (Aβ) deposition is the most important pathological feature of AD (Arbor et al., 2016). Estrogen has a negative regulatory effect on Aβ in the brain by activating Aβ degrading enzymes through estrogen receptors and finally promotes the degradation of Aβ (Liang et al., 2010). In OVX rats, Li et al. discovered that the expression levels of Aβ and its substrate synthetic amyloid precursor protein (APP) were significantly increased, and Aβ was mainly located in osteocyte membrane, plasma cells, and extracellular matrix, while APP was mainly located in the bone cell membrane (Li et al., 2014). Therefore, it is suggested that cognitive degradation and BMD reduction are both closely related to the lack of estrogen.

### Wnt Signaling Pathway

Wnt protein is a secretory morphogen needed for the basic development of many different tissues, which participates in axonal guidance, neuroblastoma migration, and neuronal differentiation in the brain (Steinhart and Angers, 2018). The classical Wnt pathway activates transcriptional cofactor β-catenin, which is the main driving factor of bone formation and the mediator of synaptogenesis and neurogenesis in the brain (Ross et al., 2018). Studies have shown that the cellular signal cascade of the Wnt signal pathway is closely related to bone loss and neuropathology. Christine et al. found that the expression of osteogenic genes in female and male htau mice was significantly lower than that in normal mice, indicating that their bone remodeling was impaired. Furthermore, genes in the Wnt/β-catenin signal were changed in the bone and brain of the htau mice, suggesting that Wnt pathway was inhibited (Dengler-Crish et al., 2018). Dickkopf1 (DKK1) is an antagonist of the Wnt signaling pathway, which can bind to the Wnt co-receptor of the low-density lipoprotein-associated receptor protein (LRP5/6). DKK1 inhibits bone formation by competing with Wnt to bind with LRP5/6 and block Wnt/β-catenin (Macdonald et al., 2009). By studying the relationship between serum DKK1 and BMD, Joseph et al. found that the concentration of serum DKK1 in the osteoporosis mice was significantly higher than that in normal mice, suggesting a negative correlation between DKK1 and bone mass (Butler et al., 2011). As for the mechanism, on the one hand, blocking of DKK1 can promote the differentiation of osteoblasts (OB) and bone formation (Yaccoby et al., 2007), and on the other hand, DKK1 can also promote the maturation and differentiation of osteoclasts (OC) *via* upregulating osteoprotegerin (OPG)/receptor activator of nuclear factor-κB ligand (RANKL) pathway (Fujita and Janz, 2007). What is more, the inhibitory effect of DKK1 is related to the neurotoxicity of Aβ and tau protein hyperphosphorylation. In the brain of transgenic AD mice, it was found that Aβ deposition could increase the expression and secretion of DKK1 and indirectly lead to neuronal apoptosis. Activation of the Wnt pathway can reduce Aβ precursor protein lyase, while overexpression of DKK1 can inhibit tau protein hyperphosphorylation caused by the Wnt signaling pathway (Scali et al., 2006). These results suggest that overexpression of DKK1 will antagonize the Wnt signaling pathway, which thereby becomes a common risk factor for AD and OP.

### OPG/RANK/RANKL and NF-κB Signaling Pathway

OB cell lineage secretes OPG, which is the only negative regulator of OC. *In vitro*, OPG can inhibit the differentiation of OC precursor cells and the formation of mature OC cells and induce OC apoptosis. The receptor activator of nuclear factor (RANK) is the only target receptor for RANKL to stimulate OC secretion and maturation (Li et al., 2016). It specifically binds with RANKL to activate the differentiation of osteoclast precursor cells and mediate bone resorption. Moreover, RANKL is a cytokine synthesized by OB and stromal cells, which can stimulate the formation of OB cells (Hsu et al., 1999). In *in vitro* experiments, it was shown that RANKL could make osteoclast precursor cells differentiate into OC, activate mature OC in a dose-dependent manner, finally prolong its survival time, and improve bone resorption capacity. OPG and RANK have competitive binding with RANKL, and OPG has a stronger competitive binding ability to RANKL than soluble RANK. On the one hand, OPG can promote OC apoptosis and terminate bone resorption by inhibiting the differentiation and maturation of OC. On the other hand, OPG can promote bone formation by nearly contacting OB. *In vivo* experiments have confirmed that OPG knockout mice had severe osteoporosis, while overexpression of OPG led to severe osteopetrosis (Bucay et al., 1998). Meanwhile, RANK is a signal complex with multiple downstream pathways. The binding of RANK and RANKL leads to the recruitment of tumor necrosis factor associated factor 6 (TRAF6) and rapidly activates the nuclear factor-κB (NF-κB) signaling pathway, thus initiating OC-specific gene transcription (van Dam et al., 2019). Long-term exposure to an inflammatory environment can significantly activate NF-κB and inhibit the function of OB. The activation of the NF-κB pathway is also related to neurofibrillary tangles (NFTs) and extracellular Aβ deposition. The increase in inflammatory factors in the AD brain will promote the degradation of APP to produce Aβ. Then, Aβ activates NF-κB that mediates the expression of inflammatory factors, which act on APP again to produce Aβ. This cascade reaction is critical in the pathogenesis of AD (June 2011). It was indicated that the inhibition of NF-κB could significantly decrease the inflammatory response caused by Aβ and reduce the damage of inflammatory factors to neuronal cells, as well as the deposition of NFTs and Aβ. Li et al. found that Aβ had no effect on RANKL-mediated OC production in rat bone marrow mononuclear cells, but it could promote bone resorption. At the molecular level, Aβ can improve the activity of NF-κB, activate the phosphorylation of extracellular signal-regulated kinase (ERK), and stimulate calcium ion oscillations, thus causing the upregulation of nuclear factor expression in OC-activated T cells (Li et al., 2016). All the above studies suggest that NF-κB can not only regulate the proliferation, differentiation, and apoptosis of OB and OC through OPG/RANK/RANKL axis system but also mediate inflammatory factors on APP to produce Aβ. This indicates that the activation of the NF-κB and OPG/RANK/RANKL signaling pathway can lead to AD and OP.

### Amyloid Precursor Protein

Amyloid precursor protein (APP) is a membrane intrinsic protein expressed in various tissues and concentrated in the synapse of neurons. *In vivo*, APP is cleaved by β-site amyloid precursor protein cleavage enzyme (BACE1) to produce soluble APP (sAPP)β, which can bind to death receptor 6 to initiate apoptosis, and further trigger axonal pruning and neuronal death. After being cleaved by the BACE1 enzyme, γ secretase can cleave the remaining fragments of APP to produce the Aβ peptide (Corbett and Hooper, 2018; Guo et al., 2021). When Aβ transport disorders occur through the blood–brain barrier, Aβ oligomers are formed and deposited, resulting in neurotoxicity and neurological dysfunction (Magzoub, 2020). As for *in vitro* experiment, it was found that APP and Aβ could activate OC and promote osteoclastic bone resorption. In the biopsy of bone tissues, Li et al. (2014) found that Aβ42 and APP in OP patients and OVX rats were significantly higher in mRNA and protein expression, compared with the control group, and were negatively correlated with BMD. Tg257 mice are medical Swedish APP gene mutant mice with a two-way regulatory effect on the activation of OC. Cui et al. (2011) found that Aβ oligomers and receptors for advanced glycation end products (RAGE) promote OC production in Tg257 mouse models under 4 months of age. In addition, extracellular Aβ deposition is closely related to the NF-κB signal pathway. Aβ could promote the activity of NF-κB and activate ERK phosphorylation, then upregulate the expression of nuclear factor of activated T cells cytoplasmic 1 (NFATc1) and promote the OC activity (Li et al., 2016). For AD patients, the increase in inflammatory factors in the brain promotes the degradation of APP to form Aβ. Subsequently, Aβ activates the NF-κB pathway, mediates the expression of inflammatory factors, and interacts with APP degradation to produce Aβ. This cascade reaction is crucial in the pathogenesis of AD (Li et al., 2016). Therefore, it is suggested that APP/Aβ is a common characteristic of AD and OP in the regulation of bone remodeling in AD patients.

### Vitamin D

Vitamin D deficiency is one of the risk factors inducing OP (Nanes and Kallen, 2014). Besides, although less well recognized, vitamin D deficiency is attracting more attention as a risk factor for AD. Observational studies have found that lower levels of plasma 25-hydroxy vitamin D are significantly related to increased risks of developing all-cause dementia and AD (Littlejohns et al., 2014). A current meta-analysis found a significant positive relationship between vitamin D deficiency and the risk of AD. It is especially noteworthy that moderate vitamin D deficiency was more strongly connected with the risk of AD compared with severe deficiency (Chai et al., 2019). Moreover, the connection was also investigated between AD and four previously identified single nucleotide polymorphisms related to vitamin D deficiency (Wang et al., 2010). It was found that an encoded vitamin D binding protein (DBP) played an important role in ameliorating AD (Mokry et al., 2016) *via* directly inhibiting Aβ aggregation and oligomerization, as well as improving Aβ-related neuronal injury and protecting against memory impairment in mice (Moon et al., 2013). On the other hand, vitamin D plays a critical role in regulating skeletal homeostasis both indirectly and directly. It was reported that vitamin D deficiency could lead to rickets in infants or children and induce OP in adults (Goltzman, 2018). As for the mechanism, the major effect of vitamin D is to regulate Ca^2+^ and Pi metabolism, promote intestinal Ca^2+^ and Pi absorption, induce bone calcification, and further protect against OP. These results provide converging evidence that vitamin D is a promising therapeutic agent for the treatment of AD and OP.

### Inflammation

Recent studies found that inflammatory bone diseases were a potential risk factor for AD. A reduced prevalence of AD was described in rheumatoid arthritis patients who were long-term users of nonsteroidal anti-inflammatory agents (NSAIDs). The epidemiological studies also demonstrated that NSAIDs use is a protective factor for AD onset (Mcgeer et al., 1990). Furthermore, an increased risk of cognitive impairment in the population with midlife rheumatoid arthritis was confirmed based on 21 years of follow-up concerning the connection between rheumatoid arthritis and AD in several case-control, hospitals, and register-based studies (Wallin et al., 2012). Besides, Kamer et al. (2008) has discovered that periodontitis induces systemic inflammation, which stimulates the production of Aβ and tau protein in the brain, leading to AD neuropathology. Interleukin-1β (IL-1β) is a key inducer of the pathogenesis and tissues damage observed in cases of inflammatory bone diseases, including periodontitis and rheumatoid arthritis (Zwerina et al., 2007). IL-1β impairs the migration of OB (Hengartner et al., 2013) and upregulates the RANKL expression induced by osteocytes (Kulkarni et al., 2012). The result of the systematic review and meta-analysis indicated that the peripheral levels of IL-1β were significantly elevated in AD patients compared with controls (Ng et al., 2018). Lipopolysaccharide-induced chronic systemic inflammation in mice resulted in prolonged IL-1β production and microglial activation in the brain (Puntener et al., 2012). Additionally, chronic elevation of IL-1β also appeared in these mice, and a rising expression of hippocampal APP and its proteolytic fragments were discovered, resulting in significant memory impairment in old age (Krstic et al., 2012). These results further suggest that inflammation is a vital risk factor for the development of AD and OP.

### Others

In addition to the above factors, there are some other common risk factors associated with AD and OP, such as PTH deficiency, CGRP, and ANS dysfunction. PTH is a key regulator of calcium homeostasis that has the potential to enhance bone regeneration in large bone defects, and its mechanism lies in its anabolic effect on bone. Daily injections of PTH are an effective treatment for OP approved by the US Food and Drug Administration (FDA) that results in increases in both BMD and bone volume (Wojda and Donahue, 2018). The connections between PTH and AD have been reported in a few prospective and case-control studies. In a prospective cohort study, high PTH concentrations were significantly and positively associated with risks of vascular dementia (Hagstrom et al., 2014). Another prospective study discovered that elevated PTH concentrations were connected with a significantly increasing risk of all-cause dementia during the 1-year and 5-year follow-up (Bjorkman et al., 2010). CGRP is a 37 amino acid regulatory neuropeptide resulting from the different merging of the CGRP gene, which has various physiological effects. However, this peptide affects inducing neuroinflammation in neurologic disorders (Malhotra, 2016). CGRP inhibition was also involved in the infiltration of macrophages and the expression of inflammatory mediators such as IL-1β and TNF-α (Singh et al., 2017). As for *in vivo* study, it was discovered that genetic depletion of calcitonin receptors in AD mice could ameliorate mice spatial memory and hippocampal synaptic plasticity, which was connected with a significant reduction in certain characteristic brain markers that were indicative of AD pathology (Patel et al., 2021). Peripheral release of CGRP contributed to the relief of arthritic pain; nevertheless, CGRP would transform the normal to persistent joint synovitis, and the expression of CGRP receptors would upregulate the following arthritic induction (Walsh et al., 2015). Besides, ANS dysfunction is also a key pathogenic factor of AD and OP. In summary, the CGRP antagonists, PTH, and agents for regulating ANS might be a potential therapeutic target to prevent persistent inflammation and attenuate the pathological cascade of AD and OP.

## Natural Chinese Medicine

### Morindae Officinalis Radix (*Morinda officinalis* How., Ba-Ji-Tian, MO)

*Morinda officinalis* How. is a traditional natural herb in China and northeast Asia, which contains many bioactive ingredients, such as iridoid glycosides, anthraquinones, and oligosaccharides. MO is known to be beneficial to the liver, kidney, and heart channels and has the action of tonifying kidney-yang, enhancing muscles and bones, and improving blood circulation. MO is widely used in TCM to treat different diseases associated with kidney-yang deficiency, such as male impotence, spermatorrhea and premature ejaculation, female infertility, and skeletal atrophy (Cai et al., 2017). Recent studies have found that MO can strengthen sexual and reproductive functions and ameliorate AD *via* regulating the microbiota-gut-brain axis, in evidence of improving memory and learning abilities (Chen et al., 2017).

According to traditional theories of Chinese medicine, cerebral diseases can be prevented by invigorating the kidneys. Therefore, MO is widely used to treat cerebral diseases, such as AD. A recent study revealed that oligosaccharides isolated from MO could significantly enhance the learning and memory abilities of rats with dementia and alleviate dementia symptoms in behavioral experiments. Subsequent *in vivo* experiments found that the oligosaccharides of MO could also increase the superoxide dismutase (SOD), glutathione peroxidase (GSH-Px), catalase (CAT), and acetylcholine (Ach) activities; decrease the malondialdehyde (MDA), acetylcholinesterase (AChE), neuronal apoptosis; and inhibit the expression of tau and Aβ_1-42_ (Chen et al., 2017). Specifically, oligosaccharide monomers, such as Bajijiasu and nystose, exhibit various biological activities, including neuroprotective, antidementia, antiosteoporosis, and antidepressant effects (Zhang et al., 2018). MO aqueous extracts could also ameliorate learning and memory impairment in AD mice. After the administration of MO aqueous extracts for 4 weeks, it was observed that MO significantly improved the learning and memory ability in a water maze test, significantly reduced the activity of aldose reductase, decreased the serum advanced glycosylation end products (AGEs) and receptor for advanced glycosylation end products (RAGEs), and then decreased the damage to brain cells (Ye et al., 2015). In D-galactose and Aβ_25-35_-induced AD rats, different doses of MO also significantly increased the antioxidant enzyme activities (SOD, GSH-Px, and CAT), neurotransmitter levels (acetylcholine, gamma-aminobutyric, and dopamine), energy metabolism (Na^+^/K^+^-ATPsae), and relative synaptophysin expression levels (Deng et al., 2020). Furthermore, there was a significant decrease in MDA levels and relative expression levels of APP, tau, and caspase-3 in AD rats after treatment with MO.

In TCM, it is considered that kidney essence deficiency plays a vital role in the development of OP, and MO is commonly used to treat bone atrophy and OP. The aqueous extract of MO has been confirmed to be effective in the treatment of postmenopausal OP, as evidenced by a clinical trial involving 50 patients (Long et al., 2013). In modern pharmacology research, it was also discovered that MO ethanol extract had protective effects on ovariectomy-induced bone loss, as evidenced by the increased tibia bone mineral content (BMC) and BMD, improved phosphorus (Pi), calcium (Ca^2+^), and OPG levels (Li et al., 2009). As for *in vitro* studies, the aqueous extracts of MO root could increase the expression of core-banding factorα 1 (Cbfα1) in bone marrow stromal cells (BMSCs) and promote the differentiation from BMSCs to OB (Wang et al., 2004). MO polysaccharide is also shown to have antiosteoporosis activities. It was discovered that MO polysaccharides could increase the BMD and BMC and decrease the cytokine levels in the serum interleukin-6 (IL-6) and tumor necrosis factor-α (TNF-α) of OVX rats after 30 days of oral administration (Mengyong et al., 2008). MO anthraquinone could also increase the mRNA expression of RANKL, alkaline phosphatase (ALP), and OPG, thereby promoting osteoblastic bone formation (Bao et al., 2011).

Taken together, these findings suggest that MO and its bioactive compounds may play an important role in the treatment and prevention of AD and OP. MO appears to provide a potentially effective treatment for AD and OP by the alleviation of learning and memory impairment and the attenuation of bone loss and trabecular microstructural degradation. Therefore, MO is a good choice for screening potential drugs to treat AD and OP.

### Eucommiae Cortex (*Eucommia ulmoides* Oliv., Du Zhong, EU)

*Eucommia ulmoides* Oliv. is an important economic plant, and its dried bark, leaves, stem, and even staminate flower are widely used in TCM. EU enjoys extensive pharmacological properties as an antioxidant, anti-inflammatory, antimicrobial, anticancer, cardioprotective, and neuroprotective agent that has been applied for the treatment of cardiovascular disease, sexual dysfunction cancer, neurological disease, rheumatoid arthritis, and osteoarthritis (He et al., 2014). The bioactive compounds of EU include lignans, iridoids, phenolics, steroids, and flavonoids, most of which have been proved to possess the ability to protect against AD and OP.

The aqueous extract of EU exhibits significant neuroprotective effects in several experimental models, thus proving to be a potential therapeutic drug in the treatment of neurodegenerative diseases, such as AD. *In vivo* studies showed that the aqueous extract of EU bark exerted a neuroprotective effect on Aβ_25–35_-induced mice and ameliorated learning and memory impairment (Kwon et al., 2011). The aqueous extract of EU significantly improved Aβ_25–35_-induced memory deficits in the Y-maze test and increased step-through latency time with Aβ_25–35_-induced learning and memory deficits in the passive avoidance test. In addition, the aqueous extract of EU also significantly inhibited AChE activity in the hippocampus and frontal cortex at 20 mg/kg concentrations and suppressed AChE activity in a dose-dependent manner, with the degrees of inhibition being 27.3% and 25.3%, respectively (Kwon et al., 2011). Moreover, aucubin in the EU appeared to ameliorate damage induced by lithium-pilocarpine in the hippocampus with status epileptics (SE), as well as reducing the number of apoptotic neurons and increasing the number of survival neurons by inducing autophagy and inhibiting necroptosis (Wang J et al., 2017). Additionally, the macranthoin G from EU effectively enhanced PC12 cells viability and increased the antioxidant enzymes, such as CAT, SOD, GSH, and GSH-Px. It also reduced intracellular reactive oxygen species (ROS), MDA content, caspase-3 activation, and PC12 cell apoptosis in *in vitro* experiments (Hu et al., 2014). These findings all revealed that EU had potential therapeutic effects on neurodegenerative diseases, such as AD.

In a rodent model of acetate-induced bone loss, EU cortex extract (EUCE) attenuated the loss of BMD of the rat’s lumbar spine and femur and restored serum Ca^2+^, Pi, ALP, osteocalcin, and RANKL to normal levels (Xiong and Zhao, 2016). Furthermore, anti-osteoclastic activity was indicated by the effect of EUCE on regulating the serum OPG/RANKL ratio to normal and significantly increased the serum Ca^2+^, Pi, ALP, and osteocalcin (Qi et al., 2019). Total lignans (TL) extracted from EU-cortex could increase BMD, biomechanical properties, and microarchitecture and inhibit the bone loss and bone turnover rate due to estrogen deficiency-induced osteoporosis in a rodent model. *In vitro* experiments also showed that TL not only increased the proliferation and differentiation of OB but also improved the formation of bone nodules (Zhang et al., 2014). The leaves and seeds of EU may also be of potential benefit for the treatment of osteoporosis. In a combined estrogen deficiency-induced osteoporosis and obesity rodent model, EU leaf extract decreased rats’ body weight and body mass index (BMI) and increased the BMD and bone strength, which appeared to be beneficial for improving bone metabolism and restoring bone loss (Zhao et al., 2020).

Taken together, it is demonstrated that EU is good medicine to treat neurodegenerative diseases and prevent bone loss for many parts of EU, including the cortex, leaf, and seed and possesses potential therapeutic benefits for the treatment of AD and OP as depicted above.

### Lycii Fructus (*Lycium barbarum* L., Gou Qi Zi, LR)

*Lycium barbarum* L., as a traditional Chinese medicinal herb and food supplement, has been used in China and other Asian countries for more than 2000 years. These berries have also become increasingly popular in western countries as an anti-aging and antioxidant product (Jin et al., 2013). The TCM theory thinks that LR can tonify the kidney, benefit essence, and strengthen the activity of the waist and knee by balancing the “yin” and “yang” in the body. LR has pharmacological properties as an anti-aging, antioxidant, anticancer, antifatigue, anti-osteoporosis, and neuroprotective agent exhibiting a wide array of therapeutic effects on aging, fatigue, cancer, stroke, AD, and OP.

Aβ is the major component of senile plaques and is considered the main biomarker of AD. Studies have found that Aβ develops its toxicity to neurons and other brain tissues by increasing the N-methyl-D-aspartate receptor- (NMDAR-) mediated Ca^2+^ influx and subsequently generating ROS (Pinheiro and Faustino, 2019). LR can protect against neuronal injury induced by Aβ peptides and promote neurogenesis. The LR polysaccharide (LRP) also shows a beneficial effect on Aβ-induced caspase-3 activation and neuronal cell death (Ho et al., 2007). The alkaline extract of LR (containing LRP) could also protect neurons *via* elevating the pro-survival AKT pathway (Ho et al., 2007). The water extract of LR played its neuronal beneficial effects by inhibiting the apoptotic pathway and associated c-Jun N-terminal kinase (JNK)/double-stranded RNA-dependent protein kinase (PKP) pathway (Yu et al., 2007). Much evidence indicated that the over-activation of the glutamatergic NMDARs leads to postsynaptic Ca^2+^ overload and excitotoxicity, resulting in disturbed neuroplasticity and neuronal cell death. Ho et al. (2009) found that LRP could antagonize glutamate-induced toxicity in primary cultures of cortical neurons, with a similar function as memantine (this non-competitive NMDAR antagonist was approved by US and European Food and Drug Administrations to be widely used in clinical trials to treat AD). LRP can also inhibit caspase-3 activation, decrease the generation of ROS, and exert an antioxidant effect. In an amyloid precursor protein/presenilin 1 (APP/PS1) double-transgenic mice model, LR water extracts strongly ameliorated the learning and memory impairments of mice after 2-week administration, which was demonstrated by the Morris water maze test (Zhang et al., 2013). These findings confirmed the protective effects of LR on AD, and the mechanism might be related to the clearance of Aβ.

LR has also shown a potent anti-OP effect in *in vivo* and *in vitro* experiments. After oral administration with 1 and 100 mg/kg LR extracts for 6 weeks, OVX rats exhibited an enhancement of BMD and BMC, decreased serum osteocalcin levels, and a recovery of the calcium levels (Kim et al., 2017). In addition, LRP could reverse palmitate-induced apoptosis in MC3T3-E1 cells by inhibiting the activation of caspase-12 and the phosphorylation level of JNK (Jing and Jia, 2018). In addition, the extract of LR cortex could also prevent BMD loss in OVX mice, improve cellular proliferation and differentiation, and increase ALP activity and mRNA expression of OB marker runt-related transcription factor 2 (Runx2) in *in vitro* experiment (Park et al., 2014). Taken together, the role of LR in promoting OB proliferation and differentiation is beyond doubt.

Collectively, as a common dual-purpose product for medicine and food, LR has the potential to develop into a drug for the prevention and treatment of AD complicated with OP for its effects on clearing Aβ and promoting OB bone formation.

### Atractylodis Macrocephalae Rhizoma (*Atractylodes macrocephala* Koidz., Bai Zhu, AM)

*Atractylodes macrocephala* Koidz., a traditional medicinal plant, is widely used as a tonic agent in China and other Asian countries. In the TCM theory, AM is considered to enter the stomach and spleen channels that can tonify “Qi” and clear damp and diuresis. AM enjoys diverse pharmacological activities, including improving gastrointestinal function, exerting anti-inflammatory, anti-oxidative, anti-aging, anti-osteoporotic, and neuroprotective activities (Zhu et al., 2018).

AM extract was found to have protective activity against AD. In the experiment of the rat passive avoidance test model, it was found to significantly prolong the step-through latency, reduce the error frequency, and decrease the AChE content after the administration of AM ethanol extract (0.5–2 mg/kg) for 15 days (Hong and Yu, 2015). This demonstrated that AM might improve memory impairment and prevent AD in aging rats, and the underlying mechanism might be associated with decreased AChE activity in the hippocampus. In a water maze experiment of AD rats induced by Aβ_1-40_, a supplement with AM biatractylolide (0.1–1.0 mg/kg) for 10 days led to a remarkably reduced swimming time and error frequency and reduced cholinesterase (ChE) content in rats, indicating that AM biatractylolide could improve the memory and learning ability of AD rats (Feng et al., 2009). In addition, AM atractylenolide also has a significant neuroprotective activity. It was discovered that the administration of AM atractylenolide could significantly enhance the cell viability in PC12 cells induced by hypoxia, calcium overloading, and excitatory amino acids (Luo and Sun, 2012). Likewise, in PC12 cells, it was found that the supplement with atractylenolide III (30 μg/ml) significantly enhanced the cell activity and suppressed apoptosis (Wang, 2012). These findings indicate that AM has a potent neuroprotective activity, especially in protecting against AD.

It was discovered that intraperitoneal injection of RANKL could rapidly reduce trabecular bone loss by stimulating OC function and differentiation rather than affecting OB formation. As for the *in vitro* experiment, AM ethanol extracts could attenuate RANKL-induced activation of the NF-κB signaling pathway and then inhibit the differentiation of OC in RANKL-induced osteoclastogenesis from OC precursors. Similar to *in vitro* results, AM also has protective effects on RANKL-induced bone loss in mice. AM inhibits RANKL-induced c-Fos and NFATc1 expression in OC precursors, thereby inhibiting OC differentiation (Ha et al., 2013). Moreover, in the mesenchymal stem cells (MSCs), the treatment with atractylenolides I and III (both are 1–300 μg/ml) can significantly improve the expression of specific chondrogenic markers and promote chondrogenic differentiation (Li X et al., 2012). Nevertheless, the anti-OP effect of other active components in AM needs to be evaluated.

Therefore, evidence suggests that AM can be used as an effective therapeutic agent for the treatment of AD and OP. Further research is needed to isolate and characterize the bioactive constituents of AM and determine the molecular mechanisms and signaling pathways that produce therapeutic effects.

### Salviae Miltiorrhizae Radix Et Rhizoma (*Salvia miltiorrhiza* Bge., Dan Shen, SM)

*Salvia miltiorrhiza* Bge. is one of the most famous TCM herbs, which has been used to treat various diseases for centuries. Its traditional efficacy is HuoXue QuYu (activate blood circulation and remove stasis), Tongjing ZhiTong (regulate menstruation and relieve pain), QingXin ChuFan (tranquilize the mind and eliminate annoyance), LiangXue XiaoYong (reduce blood flow and eliminate carbuncle). Modern pharmacological studies demonstrate that SM has diverse pharmacological activities, such as anti-oxidation, anti-inflammation, neuroprotection, anti-apoptosis, and antiosteoporosis (Sreenivasmurthy et al., 2017; Chen et al., 2019; He et al., 2019).

Accumulated evidence indicates that many ingredients of SM, such as salvianolic acids A, salvianolic acids B, cryptotanshinone tanshinone I, and tanshinone IIA, have effects on reducing cell apoptosis, inhibiting Aβ aggregation, enhancing cholinergic, and further exerting neuroprotective effects. Studies have suggested that SM extracts could significantly improve the learning and memory abilities of AD rats after being supplemented with SM for 23 consecutive days. It protected against Aβ_25-35_ induced apoptosis by downregulating APP and PS1 levels (Liu M et al., 2015). Other studies showed that AM root extracts could enhance the neurogenesis of neural stem cells and progenitor cells (Zhang XZ et al., 2016). Several reports also showed that the chemical components in SM had anti-AD effects. It was discovered that salvianolic acids A and salvianolic acids B could prevent Aβ aggregation, delay AD paralysis in *C. elegans*, and reduce ROS production to protect against Aβ induced neurotoxicity (Yuen et al., 2021). Moreover, salvianolic acid B could also improve the cholinergic damage and ameliorate Aβ-induced cognitive impairment in the AD mice model (Lee YW et al., 2013). Other research suggested that tanshinone IIA could prevent spatial learning and memory deficits in APP/PS1 mice and attenuate Aβ accumulation and neuronal loss (Ding et al., 2020).

In TCM, OP is called “bone atrophy,” and its pathogenesis is related to the liver and kidney. Furthermore, SM plays an effective role in nourishing the liver and kidney. *In vivo* studies showed that SM could attenuate the unbalanced levels of serum ALP, OPG, and RANKL in OVX rats after 14 weeks of treatment. The decreased bone strength and BMD were inhibited, and the impaired bone micro-structures were improved (Liu H et al., 2018). Similarly, SM extracts could prevent trabecular bone loss and improve the serum level of OPG in OVX mice (Park et al., 2017). These results indicate that SM has an excellent anti-bone loss effect. In addition, other active components of SM, such as salvianolic acid, also have strong bone anabolism activities. It was discovered that salvianolic acid B could enhance cancellous bone formation, improve cancellous thickness, and prevent bone loss in glucocorticoid-induced OP rats. *In vitro* experiment showed that salvianolic acid B could stimulate BMSCs differentiation to OB and increase OB activity *via* increasing the secretion of ALP and osteocalcin, improving Runx2 mRNA expression, and decreasing DKK1 mRNA expression (Cui et al., 2012). Moreover, it was found that salvianolic acid A could improve fracture callus formation and micro-architecture with an accelerated mineralization rate in callus on a prednisone-induced delayed fracture union mouse model (Liu Y et al., 2018).

In conclusion, these results indicate that both AM extracts and AM active components have significant anti-AD and anti-OP potentials by attenuating Aβ accumulation and regulating OPG and Wnt pathways. However, the underlying mechanism and clinical application are not very clear, so more research is needed.

### Rehmanniae Radix (*Rehmannia glutinosa* Libosch., Di Huang, RR)

*Rehmannia glutinosa* Libosch. has been widely used in the form of decoction pieces containing dry Radix Rehmanniae and Prepared Radix Rehmanniae in clinic. RR has various pharmacological actions, such as lowering blood pressure, anti-hyperglycemia anti-inflammation, neuroprotection, improving renal function, and antiosteoporosis (Zhang ND et al., 2016). Therefore, the clinical application of RR is to treat different diseases such as renal failure, hypertension, AD, and OP.

Although RR is not indicated in the current pharmacopeia for symptoms associated with AD, it is one of the most frequently reported ingredients of multiple TCM formulas indicated for memory impairment associated with aging (May et al., 2016). In *in vivo* screening system using the *Drosophila* model of AD, the RR extract was found to have neuroprotective activities against Aβ neurotoxicity (Liu QF et al., 2015). Catalpol is one of the most active compositions of RR and has a great potential for treating AD. In AD mice, catalpol could significantly increase the activity of SOD, GSH-Px, and CAT and reduce the levels of soluble Aβ_40_ and Aβ_42_ in the cerebral cortex of the hippocampus, thus inhibiting the formation of senile plaques. Morris water maze test revealed that the time spent in the target quadrant and average number of hidden platforms could be increased after the administration of catalpol, indicating its effects on improving the learning and memory impairment of AD mice (Huang et al., 2016). Liu et al. (Liu C et al. 2018) found that the treatment of endothelial (bEND.3) cells with catalpol could prevent endothelial damage, reduce blood–brain barrier hyperpermeability induced by fibrillar Aβ_1-42_, and enhance soluble Aβ clearance, indicating that catalpol had a protective effect on Aβ_1-42_-induced blood–brain barrier leakage. In aged rats with Aβ induced nerve injury, catalpol promoted nerve recovery and improved the expression of synaptic proteins, which was important in synaptic plasticity and neuronal development (Xia et al., 2017). Wang et al. (2018) also discovered that catalpol could upregulate α-secretase expression, promote α-cleavage of APP processing, and decrease Aβ formation *via* ERK/cAMP response element-binding protein (CREB) signaling pathway in Swedish mutant APP overexpressed N2a (SweAPP N2a) cells.

In TCM, RR has the effects on promoting blood circulation, tonifying the kidney, and regulating the liver, which are closely related to the pathogenesis of OP. Studies have discovered that water extracts of RR (300 mg/kg) could significantly improve BMD in the lumbar and femurs and markedly reduce the serum ALP level in OVX rats after an 8-week treatment (Lim and Kim, 2013). Some active components in RR, such as acteoside and catalpol, also showed a potent anti-osteoporosis effect. Acteoside could reduce ROS production and inhibit OC formation and bone resorption by macrophages *via* downregulating early RANKL signaling pathways and inhibiting the expression of TNF-α, NFATc1, and c-Fos (Lee SY et al., 2013). In addition, oral administration with acteoside could also inhibit the alteration of OP biochemical markers and bone loss in OVX rats (Lee SY et al., 2013). Similarly, Gong et al. (2019) found that acteoside, echinacoside, and catalpol could improve the proliferation and differentiation of osteoblastic MC3T3-E1 cells and increase bone morphogenetic proteins 2 (BMP2), Runx2, and osterix to prompt bone formation. As for *in vivo* study, RR extract could increase ALP activity, decrease osteocalcin (OCN) levels, and improve BMD and bone microarchitecture in diabetic rats. In addition, RR water extract could increase the serum OPG level; reduce the serum of ALP, RANKL, and tartrate-resistant acid phosphatase (TRAP) in OVX rats; and increase cortical bone and epiphyseal thickness to preserve BMD and mechanical strength by regulating Wnt/β-catenin signaling pathway (Liu et al., 2019).

Altogether, RR is considered a well-tolerated herbal medicine for AD and OP with potential neurogenic and bone-protective activities *in vivo* and *in vitro*. Its mechanism is mainly related to Aβ clearance, ERK/CREB pathway regulation, and Wnt/β-catenin activation.

### Glycyrrhizae Radix Et Rhizoma (*Glycyrrhiza uralensis* Fisch., Gan Cao, GR)

*Glycyrrhiza uralensis* Fisch. is the dried root and rhizomes of *Glycyrrhiza uralensis* Fisch., *Glycyrrhiza inflata* Bat., or *Glycyrrhiza glabra* L. and has traditionally been used in the treatment of cough, influenza, and detoxification all over the world for hundreds of years. Its clinical application has attracted extensive attention worldwide (Ji et al., 2016). Recent studies have shown that GR has antioxidant, anti-AD, anti-inflammatory, and bone protective activities.

Licochalcone B is an active compound in GR and has neuroprotective activity through inhibiting Aβ_42_ self-aggregation, reducing ROS generation, and preventing H_2_O_2_-induced SH-SY5Y cell death (Cao et al., 2020). In lipopolysaccharide-treated C57BL/6 mice, oral intake of GR extract (150 mg/kg) led to a significant reduction in spatial cognitive and memory impairment, as evidenced by the T-maze novel object recognition test (Cho et al., 2018). In another study, it was discovered that GR extract and its bio-activated compound semilicoisoflavone B could decrease the secretion of Aβ through regulating BACE1 transcription factors, as well as activating receptor-γ (PPARγ) expression and inhibiting the phosphorylation of signal transducer and activator of transcription 3 (STAT3) (Gu et al., 2018). Glycyrrhizic acid, another main bioactive compound of GR, has a neuroprotective effect on scopolamine-induced cognitive impairment. The Y-maze test found that glycyrrhizic acid could significantly improve the mice cognitive impairment induced by scopolamine, decrease AChE activity, and increase SOD and CAT activity. The mechanism was *via* increasing phosphorylation of JNK and ERK protein and improving the mitogen-activated protein kinase (MAPK) pathway (Ban et al., 2020). Additionally, some active components in GR also showed potent anti-AD effects. The coumarin glycyrol in GR could significantly suppress the butyrylcholinesterase (BChE) and AChE, and another compound, liquiritigenin, effectively inhibited monoamine oxidase B (MAO-B) and monoamine oxidase A (MAO-A). These results indicate that GR can be considered a promising herb for the treatment of AD with multi-targeting activities (Jeong et al., 2020).

In the theory of TCM, GR has a strong nutritious function and is usually used as a life enhancer. Previous studies have found that the extract of GR and its active ingredients have estrogenic-like activities (Tamir et al., 2001). Di et al. confirmed that the GR extracts notably protected BMD loss in OVX rats by measuring the BMD of the total tibia and proximal tibia. This protective effect did not affect changes in histology and weight of the uterine, indicating the absence of a uterus-focused effect of GR extract (Galanis et al., 2019). Liquiritigenin is an active flavonoid extracted from GR. It was discovered that liquiritigenin could inhibit the formation of OP phenotype in glucocorticoid-induced adult zebrafish model *via* inhibiting OC activation in scales (Carnovali et al., 2020). Another active ingredient, 18β-glycyrrhetinic acid (18β-GA), could also inhibit osteoclastogenesis and RANKL-mediated OC differentiation at an early stage *in vitro* by suppressing RANK expression in preosteoclasts and blocking the binding of TNF receptor-associated factor 6 (TRAF6) and RANK, thereby leading to the inhibition of MAPK and NF-κB signaling pathways. Furthermore, 18β-GA could reduce the serum tartrate-resistant acid phosphatase 5b (TRAP5b), TNF-α, and interleukin-6 (IL-6) and increase bone matrix mineralization, which indicated that 18β-GA decreased bone loss in OVX mice *via* suppressing osteoclastogenesis (Chen et al., 2018). In addition, studies have found that ethyl acetate extract from GR could also play a similar role as estrogen and promote the proliferation of human BMSCs (Azizsoltani et al., 2018). These results indicate that GR may be a potential candidate for the prevention of OP.

These findings indicate that GR appears to provide beneficial therapeutic effects for AD and OP, including attenuating cognitive impairment and bone loss. Hence, as a potent life enhancer, GR has the potential to be developed into a drug for the prevention and treatment of AD complicated with OP.

### Cistanches Herba (*Cistanche deserticola* Ma., Rou Cong Rong, CH)

*Cistanche deserticola* Ma., a desert living plant known as desert ginseng, is of a high medicinal value. It is characterized as the major material of tonifying kidney “yang” in the TCM theory and has been widely used to ameliorate forgetfulness, improve the strength of reproduction, and develop fertility function. Modern pharmacological studies reveal that CH has neuroprotective, immunomodulatory, anti-viral, anti-inflammatory, anti-tumor, anti-bacterial, anti-oxidative, and bone-formation activities. Therefore, CH is widely used in Chinese medicine prescriptions for treating various diseases, such as dementia, aging, and OP (Jiang et al., 2016; Fu et al., 2018; Fan et al., 2019).

Previous studies confirmed that CH extracts or its components exerted a potential neuroprotective effect against AD. Zhou et al. (2019) found that the CH extract had antiapoptotic and antioxidant stress effects *via* inhibiting Aβ deposition and tau protein hyperphosphorylation and promoting synapse protection. Chao et al. (2019) also discovered that the CH extract protected against AD by inhibiting Aβ peptide aggregation and deposition. In a clinical trial, AD patients were treated with CH capsules for 48 weeks. The results showed that the levels of TNF-α, total-tau, and IL-1β were significantly reduced, and volume changes of the hippocampus slowed down, indicating that CH could ameliorate cognitive and independent living ability through inhibiting the expression of TNF-α, total-tau, and IL-1β in the cerebrospinal fluid of AD patients (Li et al., 2015). Acteoside is one of the active phenylethanoid glycosides in CH and is known to have neuroprotective and antioxidant effects. In the AD mice model of senescence induced by a combination of AlCl_3_ and D-galactose, it was discovered that acteoside could shorten the latency of step down, reduce the number of errors, and decrease the activity of nitric oxide synthase and caspase-3 (Peng et al., 2015). Similarly, CH decoction could also improve the mice’s spatial learning and memory in the Morris water maze test by decreasing monoamine oxidase and increasing dopamine in the brain (Wang D et al., 2017).

In OVX rats, it was found that the BMD and BMC were significantly improved after treatment with CH extracts, and serum ALP and SOD CAT GSH-Px were also markedly reduced (Liang et al., 2011), suggesting that CH could reverse the bone loss and prevent postmenopausal osteoporosis. Further investigation indicated that the molecular mechanism that CH reduced bone degeneration and regulated involved bone metabolism genes, including Smad1, Smad5, TGF-β-inducible early gene 1 (TIEG1), and transforming growth factor-β1 (TGF-β1) (Liang et al., 2013). *In vitro* experiments also demonstrated that CH extracts could enhance the BMP2, osteopontin (OPN), and ALP expression and induce the OB differentiation, maturation, and bone mineralization by regulating ERK, JNK, NF-κB, and p38 (MAPK) pathways (Li TM et al., 2012). Additionally, CH polysaccharide enhanced the expression of antioxidant enzymes and reduced the RANKL-induced ROS overproduction to decrease the OC differentiation and inhibit bone resorption (Song et al., 2018).

All of these studies demonstrate the efficacy and therapeutic potential of CH in the treatment of neurodegenerative damage and bone loss. Further study is necessary to extensively characterize the bioactive effects of CH that may render better therapeutic strategies for the treatment of AD and OP.

## Discussion

In summary, with the increasingly aging population worldwide, dementia and osteoporotic fracture have become a major health and social issues. The side effects of existing clinical drugs have prompted researchers to study natural therapeutic compounds for their effectiveness and safety in the treatment of AD and OP, with fewer adverse side effects.

OP and hip fracture are commonly observed complications seen in patients with AD. Although the mechanisms underlying this association remain poorly understood, emerging evidence supports the view that AD risk genes may also be a risk factor for osteoporosis and that AD and OP may share conserved oxidative stress-driven pathogenic mechanisms (Weller and Schatzker, 2004). In addition, the pathogenesis of AD and OP is complicated in terms of occurrence, development, and progression, including estrogen deficiency; Aβ deposition; and the dysregulation of Wnt, RANKL, and NF-κB signaling pathways. Thus, the combination of multi-component and multi-target targeted therapy may be of great significance.

TCM herbs may contain effective components for the treatment of AD and OP, and this review summarizes current evidence of their potential bio-pharmacological effects and possible mechanisms. A summary of natural herbs against AD and OP *in vivo* and *in vitro* is shown in Table 1 and Table 2, summarizing the change in related indicators of each drug in *in vivo* and *in vitro* experiments.

**TABLE 1:** Summary of *in vivo* studies for the anti-Alzheimer’s disease and antiosteoporotic effects of natural Chinese medicine.

TCM	Animal model	Drug	Beneficial effects	Ref.
Morindae Officinalis Radix	AD rats	Fructo-oligosaccharides	(+) Learning and memory abilities, serum SOD, GSH-Px, CAT, and Ach	Chen et al. (2017)
			(−) Serum MDA, AChE, neuronal apoptosis, the expression of Tau and Aβ1-42	
	AD mice	Extract	(+) Learning and memory ability	Ye et al. (2015)
			(−) AR, AGEs, and RAGEs	
	AD rats	Extract	(+) Level of SOD, GSH-Px, and CAT	Deng et al. (2020)
			(−) Level of APP, tau, and caspase-3	
	OVX rats	Ethanol extract	(+) BMD and BMC, the level of P, Ca, and OPG	Li et al. (2009)
			(−) Level of serum DPD/Cr, TRAcP, and ACTH	
	OVX rats	Polysaccharides	(+) BMD and BMC	Mengyong et al. (2008)
			(−) Serum IL-6 and TNF-α	
Eucommiae Cortex	AD mice	Water extract	(+) Learning and memory	Kwon et al. (2011)
			(−) AChE	
	SE rats	Aucubin	(−) Neuronal apoptosis	Wang J et al. (2017)
	SOP rats	Extract	(+) BMD, serum Ca2+, P, ALP, osteocalcin, and RANKL	Xiong and Zhao (2016)
	OP rats	Polysaccharides	(+) Lumbar spine and femur BMD, serum Ca, P, ALP, osteocalcin, and RANKL	Qi et al. (2019)
	OVX rats	Total lignans	(+) BMD, bone biomechanical properties, microarchitecture	Zhang et al. (2014)
			(−) Bone loss, bone turnover rate	
	SAMP6	Extract	(+) BMD, bone strength	Zhao et al. (2020)
			(−) Weight, BMI, bone loss	
Lycii Fructus	APP/PS1	Extract	(−) Learning and memory impairment	Zhang et al. (2013)
	OVX mice	Extract	(+) BMD, BMC, serum Ca2+	Kim et al. (2017)
			(−) Serum osteocalcin	
	OVX mice	Extract	(−) BMD loss	Park et al. (2014)
Atractylodis Macrocephalae Rhizoma	Aging mice	Extract	(−) Memory impairment, AChE	Hong and Yu (2015)
	AD rats	Biatractylolide	(+) Memory and learning ability	Feng et al. (2009)
	Heathy mice	Extract	(−) Bone loss	Ha et al. (2013)
Salviae Miltiorrhizae Radix Et Rhizoma	AD rats	Extract	(+) Learning and memory ability	Liu M et al. (2015)
			(−) APP, PS1	
	AD *C. elegans*	Salvianolic acids A, salvianolic acids B	(−) Aβ aggregating, ROS	Yuen et al. (2021)
			(+) AD paralysis	
	AD mice	Salvianolic acids B	(−) Cholinergic damage, cognitive impairment	Lee YW et al. (2013)
	APP/PS1 mice	Tanshinone IIA	(−) Spatial learning and memory deficit, Aβ accumulation, neuronal loss	Ding et al. (2020)
	OVX rats	Extract	(+) Serum ALP, OPG, RANKL, BMD, bone strength	Liu H et al. (2018)
			(−) Bone microstructures impairment	
	OVX mice	Extract	(+) Serum OPG	Park et al. (2017)
			(−) Trabecular bone loss	
	OP rats	Salvianolic acid B	(+) Cancellous bone formation, cancellous thickness	Cui et al. (2012)
			(−) Bone loss	
	Heathy mice	Salvianolic acid A	(+) Fracture callus formation and micro-architecture, mineralization rate	Liu Y et al. (2018)
Rehmanniae Radix	AD mice	Catalpol	(+) SOD, GSH-Px, and CAT activity	Huang et al. (2016)
			(−) Learning and memory impairment, senile plaques formation	
	Aged rats	Catalpol	(+) Synaptic proteins expression, nerve recovery	Liu QF et al. (2018)
	OVX rats	Extract	(+) Lumbar and femur BMD	Lim and Kim (2013)
			(−) ALP	
	OVX rats	Acteoside	(−) OP biochemical markers, bone loss	Lee SY et al. (2013)
	Diabetic rats	Extract	(+) ALP, OCN, BMD, bone microarchitecture	Gong et al. (2019)
	OVX rats	Extract	(+) OPG, mechanical strength, cortical bone, and epiphyseal thickness	Liu et al. (2019)
			(−) ALP, RANKL, TRAP	
Glycyrrhizae Radix Et Rhizoma	C57BL/6 mice	Extract	(−) Spatial cognitive and memory impairment	Cho et al. (2018)
	Heathy mice	Glycyrrhizic acid	(+) CAT, SOD activity	Ban et al. (2020)
			(−) AChE activity, cognitive impairment	
	OVX rats	Extract	(+) BMD	Galanis et al. (2019)
	Zebrafish	Liquiritigenin	(−) OC activation	Carnovali et al. (2020)
	OVX mice	18β-GA	(+) Bone matrix mineralization	Chen et al. (2018)
			(−) Serum TRAP5b, TNF-α and IL-6, bone loss	
Cistanches Herba	AD rats	Extract	(−) Aβ peptide aggregation and deposition	Chao et al. (2019)
	AD patients	Decoction	(+) cognitive and independent living ability	Li et al. (2015)
			(−) TNF-α, total-tau and IL-1β expression	
	AD mice	Extract	(−) Nitric oxide content, nitric oxide synthase activity	Peng et al. (2015)
	Heathy mice	Decoction	(+) Spatial learning and memory dopamine	Wang D et al. (2017)
			(−) Monoamine oxidase	
	OVX rats	Extract	(+) BMD and BMC, serum ALP, SOD, CAT, GSH-Px	Liang et al. (2011)
			(−) MDA	
	OVX rats	Extract	(−) Bone degeneration, Smad1, Smad5, TIEG1, and TGF-β1 expression	Liang et al. (2013)

**TABLE 2:** Summary of *in vitro* studies for the anti-Alzheimer’s disease and antiosteoporotic effects of natural Chinese medicine.

TCM	Cells	Drug	Beneficial effects	Ref.
Morindae Officinalis Radix	BMSCs	Extract	(+) The expression of Cbfα1, differentiation	Wang et al. (2004)
	OBs	Anthraquinone	(+) RANKL, ALP, OPG, formation	Bao et al. (2011)
Eucommiae Cortex	PC12	Macranthoin G	(+) Cell viability, CAT, SOD, GSH, GSH-Px	Hu et al. (2014)
			(−) MDA, ROS	
	OBs	Total lignans	(+) ALP, proliferation and differentiation, bone nodules	Zhang et al. (2014)
Lycii Fructus	Neuronal	Extract	(+) Neurogenesis, AKT phosphorylation	Ho et al. (2007)
			(−) Lactate dehydrogenase, caspase-3	
	Neuronal	Polysaccharides	(−) Caspase-3, caspase-2, apoptosis, PKR phosphorylation	Yu et al. (2007)
	Cortical neuronal	Extract	(−) Caspase-3, ROS, apoptosis	Ho et al. (2009)
	MC3T3-E1	Polysaccharides	(−) Apoptosis, caspase-12, JNK phosphorylation	Jing and Jia (2018)
	OBs, MC3T3-E1	Extract	(+) Proliferation and differentiation, ALP, Runx2	Park et al. (2014)
Atractylodis Macrocephalae Rhizoma	PC12	Atractylenolide Ⅲ	(+) Cell viability	Luo and Sun, (2012)
	PC12	Atractylenolide Ⅲ	(+) Cell viability	Wang, (2012)
			(−) Cell apoptosis	
	Bone marrow cell, OBs, OCs	Extract	(−) Differentiation of OC, NF-κB activation, c-Fos, and NFATc1 expression	Ha et al. (2013)
	MSCs	Atractylenolide Ⅰ, atractylenolide Ⅲ	(+) Specific chondrogenic markers expression, chondrogenic differentiation	Li X et al. (2012)
Salviae Miltiorrhizae Radix Et Rhizoma	MSCs	Salvianolic acid B	(+) Differentiation, activity, secretion of ALP and osteocalcin, Runx2 mRNA expression	Cui et al. (2012)
			(−) DKK1 mRNA expression	
Rehmanniae Radix	bEND.3	Catalpol	(+) Soluble Aβ clearance	Liu C et al. (2018)
			(−) Endothelial damage, blood–brain barrier leakage	
	SweAPP N2a	Catalpol	(+) α-Secretase expression, APP processing α-cleavage	Wang et al. (2018)
			(−) Aβ formation	
	BMMs	Acteoside	(−) ROS, OC formation, bone resorption, TNF-α, NFATc1, and c-Fos expression	Lee SY et al. (2013)
	MC3T3-E1	Extract	(+) proliferation and differentiation, BMP2, Runx2, osterix	Gong et al. (2019)
Glycyrrhizae Radix Et Rhizoma	SH-SY5Y	Licochalcone B	(−) Aβ42 self-aggregation, ROS, cell death	Cao et al. (2020)
	SweAPP	Extract and semilicoisoflavone B	(+) PPARγ expression	Gu et al. (2018)
			(−) Aβ secretion, STAT3 phosphorylation	
	BMSCs	18β-GA	(−) Differentiation, RANK expression	Chen et al. (2018)
	BMSCs	Extract	(+) Proliferation	Azizsoltani et al. (2018)
Cistanches Herba	OBs	Extract	(+) BMP-2, OPN and ALP expression, differentiation, maturation, bone mineralization	Li TM et al. (2012)
	OCs	Polysaccharide	(+) Antioxidant enzymes expression	Song et al. (2018)
			(−) Differentiation, bone resorption	

According to the clinical experience and the TCM theory, Chinese herbs are divided into different categories with special functions. Because of their effects on tonifying the kidney, some of them are classic and specific drugs for improving the impairment of the brain and bone. In addition, these herbs can not only treat diseases but also have a certain preventive effect. However, the development of AD and OP remains very complex in elderly people and postmenopausal women. The mechanism of natural Chinese herbs for the treatment of AD and OP has not been fully clarified. Therefore, further study on the isolation and characterization of bioactive components against AD and OP from classic and specific drugs is necessary to widely profile components for pharmacological uses, especially their efficacy, safety, and potential interaction with other medications. The research to determine the special and targeted cellular and molecular mechanisms of natural Chinese medicine components should develop their potential application in the treatment of AD and OP as an effective and safe alternative to the major treatment strategy or in combination with current pharmacological therapy. Besides, few high-quality clinical research studies have presented against the AD and OP effects of components with known structures. Because there maybe unknown chemical interactions between various drugs and non-specific components in traditional formulas, the clinical drug research results still have deficiencies and limitations. Therefore, more high-quality clinical research studies of TCM are required in the future to explore the anabolic and anti-metabolic effects.

## Conclusion

As common age-related diseases, AD and OP have similarities in pathological characteristics and pathogenesis. Recent *in vivo* and *in vitro* findings indicate that TCM herbs may well be effective for the treatment of AD complicated with OP. Further study is required to assure the efficacy, safety, and specificity of the components in TCM in order to tap its therapeutic potential. Therefore, more high-quality clinical studies of these natural drugs are required to provide more powerful evidence for candidate drugs so that they can be used as beneficial and safer against AD and OP applications.

## Abbreviations

Aβ, β-amyloid; AD, Alzheimer’s disease; AGEs, advance glycosylation end products; APP, amyloid precursor protein; AR, aldose reductase; BACE1, β-site amyloid precursor protein cleavage enzyme 1; BMD, bone mineral density; BMI, body mass index; BMP2, bone morphogenetic proteins 2; BMSCs, bone marrow stromal cells; CAT, catalase; Cbfα1, core-banding factor α-1; ChE, cholinesterase; DKK1, Dickkopf1; ERK, extracellular signal-regulated kinase; GSH-Px, glutathione peroxidase; IL-6, interleukin-6; JNK, c-Jun N-terminal kinase; MAO, monoamine oxidase; MDA, malondialdehyde; MSCs, mesenchymal stem cells; NFATc1, nuclear factor of activated T cells cytoplasmic 1; NF-κB, nuclear factor-κB; NFTs, neurofibrillary tangles; NMDAR, N-methyl-D-aspartate receptor; OB, osteoblasts; OC, osteoclasts; OCN, osteocalcin; OP, osteoporosis; OPG, osteoprotegerin; OPN, osteopontin; PPARγ, activate receptor-γ; RAGEs, receptor for advanced glycosylation end products; RANK, receptor activator of nuclear factor; RANKL, receptor for activator nuclear factor-κB ligand; ROS, reactive oxygen species; Runx2, runt-related transcription factor 2; STAT3, signal transducer and activator of transcription 3; TCM, traditional Chinese medicine; TL, total lignans; TNF-α, tumor necrosis factor-α; TRAF6, tumor necrosis factor associated factor 6.

## References

[B1] Abrahamsen B. Osmond C. Cooper C. (2015). Life Expectancy in Patients Treated for Osteoporosis: Observational Cohort Study Using National Danish Prescription Data. J. Bone Miner. Res. 30 (9), 1553–1559. 10.1002/jbmr.2478 25663501

[B2] Amouzougan A. Lafaie L. Marotte H. Dẻnariẻ D. Collet P. Pallot-Prades B. (2017). High Prevalence of Dementia in Women with Osteoporosis. Jt. Bone Spine 84 (5), 611–614. 10.1016/j.jbspin.2016.08.002 27697401

[B3] Arbor S. C. Lafontaine M. Cumbay M. (2016). Amyloid-beta Alzheimer Targets - Protein Processing, Lipid Rafts, and Amyloid-Beta Pores. Yale J. Biol. Med. 89 (1), 5–21. 27505013 PMC4797837

[B4] Armas L. A. Recker R. R. (2012). Pathophysiology of Osteoporosis: New Mechanistic Insights. Endocrinol. Metab. Clin. North. Am. 41 (3), 475–486. 10.1016/j.ecl.2012.04.006 22877425

[B5] Aspray T. J. Hill T. R. (2019). Osteoporosis and the Ageing Skeleton. Subcell Biochem. 91, 453–476. 10.1007/978-981-13-3681-2_16 30888662

[B6] Alzheimer’s Disease International, McGill University (2021). Available at: https://www.alzint.org/resource/world-alzheimer-report-2021/ (Accessed September 21, 2021).

[B7] Osteoporosis and Bone Mineral Disease Branch of Chinese Medical Association (2019). Epidemiological Survey of Osteoporosis in China and “Healthy Bones” Special Action Results Released. Chin. J. Osteoporos. Bone Mineral Res. 12 (04), 317–318.

[B8] Azizsoltani A. Piri K. Behzad S. Soleimani M. Nekouei M. Mahmoudi Z. (2018). Ethyl Acetate Extract of Licorice Root (Glycyrrhiza Glabra) Enhances Proliferation and Osteogenic Differentiation of Human Bone Marrow Mesenchymal Stem Cells. Iran. J. Pharm. Res. 17 (3), 1057–1067. 30127828 PMC6094414

[B9] Ban J. Y. Park H. K. Kim S. K. (2020). Effect of Glycyrrhizic Acid on Scopolamine-Induced Cognitive Impairment in Mice. Int. Neurourol J. 24 (Suppl. 1), S48–S55. 10.5213/inj.2040154.077 32482057 PMC7285697

[B10] Bao L. Qin L. Liu L. Wu Y. Han T. Xue L. (2011). Anthraquinone Compounds from Morinda Officinalis Inhibit Osteoclastic Bone Resorption In Vitro. Chem. Biol. Interact 194 (2-3), 97–105. 10.1016/j.cbi.2011.08.013 21945525

[B11] Björkman M. P. Sorva A. J. Tilvis R. S. (2010). Does Elevated Parathyroid Hormone Concentration Predict Cognitive Decline in Older People? Aging Clin. Exp. Res. 22 (2), 164–169. 10.1007/BF03324791 19934619

[B12] Bucay N. Sarosi I. Dunstan C. R. Morony S. Tarpley J. Capparelli C. (1998). Osteoprotegerin-Deficient Mice Develop Early Onset Osteoporosis and Arterial Calcification. Genes Dev. 12 (9), 1260–1268. 10.1101/gad.12.9.1260 9573043 PMC316769

[B13] Butler J. S. Murray D. W. Hurson C. J. O'Brien J. Doran P. P. O'Byrne J. M. (2011). The Role of Dkk1 in Bone Mass Regulation: Correlating Serum Dkk1 Expression with Bone mineral Density. J. Orthop. Res. 29 (3), 414–418. 10.1002/jor.21260 20939046

[B14] Cai H. Wang Y. He J. Cai T. Wu J. Fang J. (2017). Neuroprotective Effects of Bajijiasu against Cognitive Impairment Induced by Amyloid-β in APP/PS1 Mice. Oncotarget 8 (54), 92621–92634. 10.18632/oncotarget.21515 29190943 PMC5696209

[B15] Cao Y. Xu W. Huang Y. Zeng X. (2020). Licochalcone B, a Chalcone Derivative from Glycyrrhiza Inflata, as a Multifunctional Agent for the Treatment of Alzheimer's Disease. Nat. Prod. Res. 34 (5), 736–739. 10.1080/14786419.2018.1496429 30345819

[B16] Carnovali M. Banfi G. Mariotti M. (2020). Liquiritigenin Reduces Osteoclast Activity in Zebrafish Model of Glucocorticoid-Induced Osteoporosis. J. Pharmacol. Sci. 143 (4), 300–306. 10.1016/j.jphs.2020.06.001 32534995

[B17] Chai B. Gao F. Wu R. Dong T. Gu C. Lin Q. (2019). Vitamin D Deficiency as a Risk Factor for Dementia and Alzheimer's Disease: an Updated Meta-Analysis. BMC Neurol. 19 (1), 284. 10.1186/s12883-019-1500-6 31722673 PMC6854782

[B18] Chao C. L. Huang H. W. Huang H. C. Chao H. F. Yu S. W. Su M. H. (2019). Inhibition of Amyloid Beta Aggregation and Deposition of Cistanche Tubulosa Aqueous Extract. Molecules 24 (4). 10.3390/molecules24040687 PMC6412839 30769881

[B19] Chen D. Yang X. Yang J. Lai G. Yong T. Tang X. (2017). Prebiotic Effect of Fructooligosaccharides from Morinda Officinalis on Alzheimer's Disease in Rodent Models by Targeting the Microbiota-Gut-Brain Axis. Front. Aging Neurosci. 9, 403. 10.3389/fnagi.2017.00403 29276488 PMC5727096

[B20] Chen P. Li Z. Hu Y. (2016). Prevalence of Osteoporosis in China: a Meta-Analysis and Systematic Review. BMC Public Health 16 (1), 1039. 10.1186/s12889-016-3712-7 27716144 PMC5048652

[B21] Chen X. Yu J. Zhong B. Lu J. Lu J. J. Li S. (2019). Pharmacological Activities of Dihydrotanshinone I, a Natural Product from Salvia Miltiorrhiza Bunge. Pharmacol. Res. 145, 104254. 10.1016/j.phrs.2019.104254 31054311

[B22] Chen X. Zhi X. Yin Z. Li X. Qin L. Qiu Z. (2018). 18β-Glycyrrhetinic Acid Inhibits Osteoclastogenesis In Vivo and In Vitro by Blocking RANKL-Mediated RANK-TRAF6 Interactions and NF-Κb and MAPK Signaling Pathways. Front. Pharmacol. 9, 647. 10.3389/fphar.2018.00647 29973878 PMC6019442

[B23] Chen Y. H. Lo R. Y. (2017). Alzheimer's Disease and Osteoporosis. Ci Ji Yi Xue Za Zhi 29 (3), 138–142. 10.4103/tcmj.tcmj_54_17 28974906 PMC5615992

[B24] Cho M. J. Kim J. H. Park C. H. Lee A. Y. Shin Y. S. Lee J. H. (2018). Comparison of the Effect of Three Licorice Varieties on Cognitive Improvement via an Amelioration of Neuroinflammation in Lipopolysaccharide-Induced Mice. Nutr. Res. Pract. 12 (3), 191–198. 10.4162/nrp.2018.12.3.191 29854324 PMC5974064

[B25] Corbett N. J. Hooper N. M. (2018). Soluble Amyloid Precursor Protein α: Friend or Foe? Adv. Exp. Med. Biol. 1112, 177–183. 10.1007/978-981-13-3065-0_13 30637698

[B26] Cui L. Li T. Liu Y. Zhou L. Li P. Xu B. (2012). Salvianolic Acid B Prevents Bone Loss in Prednisone-Treated Rats through Stimulation of Osteogenesis and Bone Marrow Angiogenesis. PLoS One 7 (4), e34647. 10.1371/journal.pone.0034647 22493705 PMC3321026

[B27] Cui S. Xiong F. Hong Y. Jung J. U. Li X. S. Liu J. Z. (2011). APPswe/Aβ Regulation of Osteoclast Activation and RAGE Expression in an Age-dependent Manner. J. Bone Miner. Res. 26 (5), 1084–1098. 10.1002/jbmr.299 21542009 PMC3126661

[B28] Deng S. Lu H. Chi H. Wang Y. Li X. Ye H. (2020). Neuroprotective Effects of OMO within the Hippocampus and Cortex in a D-Galactose and Aβ 25-35-Induced Rat Model of Alzheimer's Disease. Evid. Based Complement. Alternat Med. 2020, 1067541. 10.1155/2020/1067541 33101436 PMC7569426

[B29] Dengler-Crish C. M. Ball H. C. Lin L. Novak K. M. Cooper L. N. (2018). Evidence of Wnt/β-Catenin Alterations in Brain and Bone of A tauopathy Mouse Model of Alzheimer's Disease. Neurobiol. Aging 67, 148–158. 10.1016/j.neurobiolaging.2018.03.021 29660685

[B30] Depypere H. Vierin A. Weyers S. Sieben A. (2016). Alzheimer's Disease, Apolipoprotein E and Hormone Replacement Therapy. Maturitas 94, 98–105. 10.1016/j.maturitas.2016.09.009 27823753

[B31] Ding B. Lin C. Liu Q. He Y. Ruganzu J. B. Jin H. (2020). Tanshinone IIA Attenuates Neuroinflammation via Inhibiting RAGE/NF-κB Signaling Pathway In Vivo and In Vitro. J. Neuroinflammation 17 (1), 302. 10.1186/s12974-020-01981-4 33054814 PMC7559789

[B32] Eiken P. Vestergaard P. Jensen J. E. (2014). Hormone Replacement Therapy as Primary Prevention? Ugeskr Laeger 176 (6). 25096207

[B33] Éric L. (2020). Epidemiology of Osteoporosis. Rev. Prat 70 (9), 1018–1022. 33739765

[B34] Fan X. Chen Y. Li L. Wang Y. Zhang Y. Lu S. (2019). Efficacy of Chinese Herb Cistanche Yishen Granules in Treatment of Tinnitus for Patients with Chronic Nephritis. J. Cel. Biochem. 120 (4), 5505–5509. 10.1002/jcb.27833 30474893

[B35] Feng X. Wang Z. Lin Y. Zhou Y. Liu Y. Yang H. (2009). Effects of Biatractylolide on the AD Rats Induced by Aβ_(1-40). Chin. Pharmacol. Bull. 25, 949–951. 10.3321/j.issn:1001-1978.2009.07.029

[B36] Fu Z. Fan X. Wang X. Gao X. (2018). Cistanches Herba: An Overview of its Chemistry, Pharmacology, and Pharmacokinetics Property. J. Ethnopharmacol. 219, 233–247. 10.1016/j.jep.2017.10.015 29054705

[B37] Fujita K. Janz S. (2007). Attenuation of WNT Signaling by DKK-1 and -2 Regulates BMP2-Induced Osteoblast Differentiation and Expression of OPG, RANKL and M-CSF. Mol. Cancer 6, 71. 10.1186/1476-4598-6-71 17971207 PMC2173906

[B38] Galanis D. Soultanis K. Lelovas P. Zervas A. Papadopoulos P. Galanos A. (2019). Protective Effect of Glycyrrhiza Glabra Roots Extract on Bone mineral Density of Ovariectomized Rats. Biomedicine (Taipei) 9 (2), 8. 10.1051/bmdcn/2019090208 31124454 PMC6533940

[B39] Goltzman D. (2018). Functions of Vitamin D in Bone. Histochem. Cel Biol. 149 (4), 305–312. 10.1007/s00418-018-1648-y 29435763

[B40] Gong W. Zhang N. Cheng G. Zhang Q. He Y. Shen Y. (2019). Rehmannia Glutinosa Libosch Extracts Prevent Bone Loss and Architectural Deterioration and Enhance Osteoblastic Bone Formation by Regulating the IGF-1/PI3K/mTOR Pathway in Streptozotocin-Induced Diabetic Rats. Int. J. Mol. Sci. 20 (16). 10.3390/ijms20163964 PMC6720794 31443143

[B41] Gu M. Y. Chun Y. S. Zhao D. Ryu S. Y. Yang H. O. (2018). Glycyrrhiza Uralensis and Semilicoisoflavone B Reduce Aβ Secretion by Increasing PPARγ Expression and Inhibiting STAT3 Phosphorylation to Inhibit BACE1 Expression. Mol. Nutr. Food Res. 62 (6), e1700633. 10.1002/mnfr.201700633 29143445

[B42] Guo Y. Wang Q. Chen S. Xu C. (2021). Functions of Amyloid Precursor Protein in Metabolic Diseases. Metabolism 115, 154454. 10.1016/j.metabol.2020.154454 33248065

[B43] Ha H. An H. Shim K. S. Kim T. Lee K. J. Hwang Y. H. (2013). Ethanol Extract of Atractylodes Macrocephala Protects Bone Loss by Inhibiting Osteoclast Differentiation. Molecules 18 (7), 7376–7388. 10.3390/molecules18077376 23884114 PMC6269826

[B44] Hagström E. Kilander L. Nylander R. Larsson E. M. Michaëlsson K. Melhus H. (2014). Plasma Parathyroid Hormone Is Associated with Vascular Dementia and Cerebral Hyperintensities in Two Community-Based Cohorts. J. Clin. Endocrinol. Metab. 99 (11), 4181–4189. 10.1210/jc.2014-1736 25140397

[B45] He J. Li X. Wang Z. Bennett S. Chen K. Xiao Z. (2019). Therapeutic Anabolic and Anticatabolic Benefits of Natural Chinese Medicines for the Treatment of Osteoporosis. Front. Pharmacol. 10, 1344. 10.3389/fphar.2019.01344 31824310 PMC6886594

[B46] He X. Wang J. Li M. Hao D. Yang Y. Zhang C. (2014). Eucommia Ulmoides Oliv.: Ethnopharmacology, Phytochemistry and Pharmacology of an Important Traditional Chinese Medicine. J. Ethnopharmacol. 151 (1), 78–92. 10.1016/j.jep.2013.11.023 24296089

[B47] Henderson V. W. (2006). Estrogen-containing Hormone Therapy and Alzheimer's Disease Risk: Understanding Discrepant Inferences from Observational and Experimental Research. Neuroscience 138 (3), 1031–1039. 10.1016/j.neuroscience.2005.06.017 16310963

[B48] Hengartner N. E. Fiedler J. Ignatius A. Brenner R. E. (2013). IL-1β Inhibits Human Osteoblast Migration. Mol. Med. 19, 36–42. 10.2119/molmed.2012.00058 23508571 PMC3646095

[B49] Ho Y. S. Yu M. S. Lai C. S. So K. F. Yuen W. H. Chang R. C. (2007). Characterizing the Neuroprotective Effects of Alkaline Extract of Lycium Barbarum on Beta-Amyloid Peptide Neurotoxicity. Brain Res. 1158, 123–134. 10.1016/j.brainres.2007.04.075 17568570

[B50] Ho Y. S. Yu M. S. Yik S. Y. So K. F. Yuen W. H. Chang R. C. (2009). Polysaccharides from Wolfberry Antagonizes Glutamate Excitotoxicity in Rat Cortical Neurons. Cell. Mol. Neurobiol. 29 (8), 1233–1244. 10.1007/s10571-009-9419-x 19499323 PMC11505788

[B51] Høgh P. (2017). Alzheimer's Disease. Ugeskr Laeger 179 (12), 1. 28330540

[B52] Hong J. Z. Yu Y. X. (2015). Effect of Ethanol Extracts of Atractylodis Macrocephalae on Memeory Impairment in Aging Mice. Dalian, China: Dalian University.

[B53] Howes M. R. Fang R. Houghton P. J. (2017). Effect of Chinese Herbal Medicine on Alzheimer's Disease. Int. Rev. Neurobiol. 135, 29–56. 10.1016/bs.irn.2017.02.003 28807163

[B54] Hsu H. Lacey D. L. Dunstan C. R. Solovyev I. Colombero A. Timms E. (1999). Tumor Necrosis Factor Receptor Family Member RANK Mediates Osteoclast Differentiation and Activation Induced by Osteoprotegerin Ligand. Proc. Natl. Acad. Sci. U S A. 96 (7), 3540–3545. 10.1073/pnas.96.7.3540 10097072 PMC22329

[B55] Hu W. Wang G. Li P. Wang Y. Si C. L. He J. (2014). Neuroprotective Effects of Macranthoin G from Eucommia Ulmoides against Hydrogen Peroxide-Induced Apoptosis in PC12 Cells via Inhibiting NF-Κb Activation. Chem. Biol. Interact 224, 108–116. 10.1016/j.cbi.2014.10.011 25451577

[B56] Huang J. Z. Wu J. Xiang S. Sheng S. Jiang Y. Yang Z. (2016). Catalpol Preserves Neural Function and Attenuates the Pathology of Alzheimer's Disease in Mice. Mol. Med. Rep. 13 (1), 491–496. 10.3892/mmr.2015.4496 26531891

[B57] Jeong G. S. Kang M. G. Lee J. Y. Lee S. R. Park D. Cho M. (2020). Inhibition of Butyrylcholinesterase and Human Monoamine Oxidase-B by the Coumarin Glycyrol and Liquiritigenin Isolated from Glycyrrhiza Uralensis. Molecules 25 (17). 10.3390/molecules25173896 PMC7504216 32859055

[B58] Ji S. Li Z. Song W. Wang Y. Liang W. Li K. (2016). Bioactive Constituents of Glycyrrhiza Uralensis (Licorice): Discovery of the Effective Components of a Traditional Herbal Medicine. J. Nat. Prod. 79 (2), 281–292. 10.1021/acs.jnatprod.5b00877 26841168

[B59] Jia J. Wei C. Chen S. Li F. Tang Y. Qin W. (2018). The Cost of Alzheimer's Disease in China and Re-estimation of Costs Worldwide. Alzheimers Dement 14 (4), 483–491. 10.1016/j.jalz.2017.12.006 29433981

[B60] Jiang Z. Wang J. Li X. Zhang X. (2016). Echinacoside and Cistanche Tubulosa (Schenk) R. Wight Ameliorate Bisphenol A-Induced Testicular and Sperm Damage in Rats through Gonad axis Regulated Steroidogenic Enzymes. J. Ethnopharmacol. 193, 321–328. 10.1016/j.jep.2016.07.033 27422164

[B61] Jin M. Huang Q. Zhao K. Shang P. (2013). Biological Activities and Potential Health Benefit Effects of Polysaccharides Isolated from Lycium Barbarum L. Int. J. Biol. Macromol. 54, 16–23. 10.1016/j.ijbiomac.2012.11.023 23200976

[B62] Jing L. Jia X. W. (2018). Lycium Barbarum Polysaccharide Arbitrates Palmitate-Induced Apoptosis in MC3T3-E1 C-ells through D-ecreasing the A-ctivation of ERS-mediated A-poptosis P-athway. Mol. Med. Rep. 17 (2), 2415–2421. 10.3892/mmr.2017.8128 29207092 PMC5783487

[B63] Jun W. (2011). Experimental Study on the Pathogenesis, Diagnosis and Treatment of Alzheimer's Disease. Wuhan, China: Huazhong University of Science and Technology.

[B64] Kamer A. R. Craig R. G. Dasanayake A. P. Brys M. Glodzik-Sobanska L. de Leon M. J. (2008). Inflammation and Alzheimer's Disease: Possible Role of Periodontal Diseases. Alzheimers Dement 4 (4), 242–250. 10.1016/j.jalz.2007.08.004 18631974

[B65] Kim M. H. Lee J. E. Lee J. S. Yang W. M. (2017). Improvement of Osteoporosis by Lycium Chinense Administration in Ovariectomized Mice. J. Chin. Med. Assoc. 80 (4), 222–226. 10.1016/j.jcma.2016.11.006 28268173

[B66] Kim M. Y. Kim K. Hong C. H. Lee S. Y. Jung Y. S. (2018). Sex Differences in Cardiovascular Risk Factors for Dementia. Biomol. Ther. (Seoul) 26 (6), 521–532. 10.4062/biomolther.2018.159 30464071 PMC6254640

[B67] Krstic D. Madhusudan A. Doehner J. Vogel P. Notter T. Imhof C. (2012). Systemic Immune Challenges Trigger and Drive Alzheimer-like Neuropathology in Mice. J. Neuroinflammation 9, 151. 10.1186/1742-2094-9-151 22747753 PMC3483167

[B68] Kulkarni R. N. Bakker A. D. Everts V. Klein-Nulend J. (2012). Mechanical Loading Prevents the Stimulating Effect of IL-1β on Osteocyte-Modulated Osteoclastogenesis. Biochem. Biophys. Res. Commun. 420 (1), 11–16. 10.1016/j.bbrc.2012.02.099 22390927

[B69] Kwon S. H. Lee H. K. Kim J. A. Hong S. I. Kim S. Y. Jo T. H. (2011). Neuroprotective Effects of Eucommia Ulmoides Oliv. Bark on Amyloid Beta(25-35)-Induced Learning and Memory Impairments in Mice. Neurosci. Lett. 487 (1), 123–127. 10.1016/j.neulet.2010.10.042 20974223

[B70] Lamichhane A. P. (2005). Osteoporosis-an Update. JNMA J. Nepal Med. Assoc. 44 (158), 60–66. 10.31729/jnma.404 16568580

[B71] Laudisio A. Fontana D. O. Rivera C. Ruggiero C. Bandinelli S. Gemma A. (2016). Bone Mineral Density and Cognitive Decline in Elderly Women: Results from the InCHIANTI Study. Calcif Tissue Int. 98 (5), 479–488. 10.1007/s00223-015-0102-6 26713334 PMC6117833

[B72] Lee D. Y. Na D. L. Seo S. W. Chin J. Lim S. J. Choi D. (2012). Association between Cognitive Impairment and Bone mineral Density in Postmenopausal Women. Menopause 19 (6), 636–641. 10.1097/gme.0b013e31823dbec7 22334060

[B73] Lee J. H. Bliwise D. L. Ansari F. P. Goldstein F. C. Cellar J. S. Lah J. J. (2007). Daytime Sleepiness and Functional Impairment in Alzheimer Disease. Am. J. Geriatr. Psychiatry 15 (7), 620–626. 10.1097/JGP.0b013e3180381521 17586786

[B74] Lee S. Y. Lee K. S. Yi S. H. Kook S. H. Lee J. C. (2013). Acteoside Suppresses RANKL-Mediated Osteoclastogenesis by Inhibiting C-Fos Induction and NF-Κb Pathway and Attenuating ROS Production. PLoS One 8 (12), e80873. 10.1371/journal.pone.0080873 24324641 PMC3851776

[B75] Lee Y. W. Kim D. H. Jeon S. J. Park S. J. Kim J. M. Jung J. M. (2013). Neuroprotective Effects of Salvianolic Acid B on an Aβ25-35 Peptide-Induced Mouse Model of Alzheimer's Disease. Eur. J. Pharmacol. 704 (1-3), 70–77. 10.1016/j.ejphar.2013.02.015 23461850

[B76] Li J J. Wang G. B. Feng X. Zhang J. Fu Q. (2016). Effect of Gallium Nitrate on the Expression of Osteoprotegerin and Receptor Activator of Nuclear factor-κB L-igand in O-steoblasts In V-ivo and In V-itro. Mol. Med. Rep. 13 (1), 769–777. 10.3892/mmr.2015.4588 26647856 PMC4686028

[B77] Li N. Qin L. P. Han T. Wu Y. B. Zhang Q. Y. Zhang H. (2009). Inhibitory Effects of morinda Officinalis Extract on Bone Loss in Ovariectomized Rats. Molecules 14 (6), 2049–2061. 10.3390/molecules14062049 19513005 PMC6254270

[B78] Li N. Wang J. Ma J. Gu Z. Jiang C. Yu L. (2015). Neuroprotective Effects of Cistanches Herba Therapy on Patients with Moderate Alzheimer's Disease. Evid. Based Complement. Alternat Med. 2015, 103985. 10.1155/2015/103985 26435722 PMC4576016

[B79] Li S. Liu B. Zhang L. Rong L. (2014). Amyloid Beta Peptide Is Elevated in Osteoporotic Bone Tissues and Enhances Osteoclast Function. Bone 61, 164–175. 10.1016/j.bone.2014.01.010 24473375

[B80] Li S S. Yang B. Teguh D. Zhou L. Xu J. Rong L. (2016). Amyloid β Peptide Enhances RANKL-Induced Osteoclast Activation through NF-Κb, ERK, and Calcium Oscillation Signaling. Int. J. Mol. Sci. 17 (10). 10.3390/ijms17101683 PMC5085715 27735865

[B81] Li T. M. Huang H. C. Su C. M. Ho T. Y. Wu C. M. Chen W. C. (2012). Cistanche Deserticola Extract Increases Bone Formation in Osteoblasts. J. Pharm. Pharmacol. 64 (6), 897–907. 10.1111/j.2042-7158.2012.01483.x 22571269

[B82] Li X. Wei G. Wang X. Liu D. H. Deng R. D. Li H. (2012). Targeting of the Sonic Hedgehog Pathway by Atractylenolides Promotes Chondrogenic Differentiation of Mesenchymal Stem Cells. Biol. Pharm. Bull. 35 (8), 1328–1335. 10.1248/bpb.b12-00265 22863933

[B83] Liang H. Yu F. Tong Z. Huang Z. (2011). Effect of Cistanches Herba Aqueous Extract on Bone Loss in Ovariectomized Rat. Int. J. Mol. Sci. 12 (8), 5060–5069. 10.3390/ijms12085060 21954345 PMC3179152

[B84] Liang H. D. Yu F. Tong Z. H. Zhang H. Q. Liang W. (2013). Cistanches Herba Aqueous Extract Affecting Serum BGP and TRAP and Bone Marrow Smad1 mRNA, Smad5 mRNA, TGF-Β1 mRNA and TIEG1 mRNA Expression Levels in Osteoporosis Disease. Mol. Biol. Rep. 40 (2), 757–763. 10.1007/s11033-012-2065-2 23232713

[B85] Liang K. Yang L. Yin C. Xiao Z. Zhang J. Liu Y. (2010). Estrogen Stimulates Degradation of Beta-Amyloid Peptide by Up-Regulating Neprilysin. J. Biol. Chem. 285 (2), 935–942. 10.1074/jbc.M109.051664 19897485 PMC2801294

[B86] Lim D. W. Kim Y. T. (2013). Dried Root of Rehmannia Glutinosa Prevents Bone Loss in Ovariectomized Rats. Molecules 18 (5), 5804–5813. 10.3390/molecules18055804 23685937 PMC6270096

[B87] Littlejohns T. J. Henley W. E. Lang I. A. Annweiler C. Beauchet O. Chaves P. H. (2014). Vitamin D and the Risk of Dementia and Alzheimer Disease. Neurology 83 (10), 920–928. 10.1212/WNL.0000000000000755 25098535 PMC4153851

[B88] Liu C. Chen K. Lu Y. Fang Z. Yu G. (2018). Catalpol Provides a Protective Effect on Fibrillary Aβ1-42 -induced Barrier Disruption in an In Vitro Model of the Blood-Brain Barrier. Phytother. Res. 32 (6), 1047–1055. 10.1002/ptr.6043 29479743

[B89] Liu C. Wang L. Zhu R. Liu H. Ma R. Chen B. (2019). Correction to: Rehmanniae Radix Preparata Suppresses Bone Loss and Increases Bone Strength through Interfering with Canonical Wnt/β-Catenin Signaling Pathway in OVX Rats. Osteoporos. Int. 30 (7), 1537–1540. 10.1007/s00198-019-05028-0 31214751

[B90] Liu H. Zhu R. Wang L. Liu C. Ma R. Qi B. (2018). Radix Salviae Miltiorrhizae Improves Bone Microstructure and Strength through Wnt/β-Catenin and Osteoprotegerin/receptor Activator for Nuclear Factor-Κb Ligand/cathepsin K Signaling in Ovariectomized Rats. Phytother. Res. 32 (12), 2487–2500. 10.1002/ptr.6188 30306652

[B91] Liu M. Guo H. Li C. Wang D. Wu J. Wang C. (2015). Cognitive Improvement of Compound Danshen in an Aβ25-35 Peptide-Induced Rat Model of Alzheimer's Disease. BMC Complement. Altern. Med. 15, 382. 10.1186/s12906-015-0906-y 26497584 PMC4619010

[B92] Liu Q. F. Lee J. H. Kim Y. M. Lee S. Hong Y. K. Hwang S. (2015). In Vivo Screening of Traditional Medicinal Plants for Neuroprotective Activity against Aβ42 Cytotoxicity by Using Drosophila Models of Alzheimer's Disease. Biol. Pharm. Bull. 38 (12), 1891–1901. 10.1248/bpb.b15-00459 26458335

[B93] Liu Y. Jia Z. Akhter M. P. Gao X. Wang X. Wang X. (2018). Bone-targeting Liposome Formulation of Salvianic Acid A Accelerates the Healing of Delayed Fracture Union in Mice. Nanomedicine 14 (7), 2271–2282. 10.1016/j.nano.2018.07.011 30076934

[B94] Long H. Ye W. M. Mai W. (2013). Effect of Medicinal Indianmulberry Root in the Treatment of Postmenopausal Osteoporosis. China Med. Pharm. 3 (14), 80–81.

[B95] Lorentzon M. Johansson H. Harvey N. C. Liu E. Vandenput L. Mccloskey E. V. (2021). Osteoporosis and Fractures in Women: the burden of Disease. Climacteric 25, 4–10. 10.1080/13697137.2021.1951206 34319208

[B96] Loskutova N. Honea R. A. Brooks W. M. Burns J. M. (2010). Reduced Limbic and Hypothalamic Volumes Correlate with Bone Density in Early Alzheimer's Disease. J. Alzheimers Dis. 20 (1), 313–322. 10.3233/JAD-2010-1364 20164583 PMC2892930

[B97] Loskutova N. Honea R. A. Vidoni E. D. Brooks W. M. Burns J. M. (2009). Bone Density and Brain Atrophy in Early Alzheimer's Disease. J. Alzheimers Dis. 18 (4), 777–785. 10.3233/JAD-2009-1185 19661621 PMC2842449

[B98] Luo L. Sun Y. (2012). Effect of Atractylenolide Ⅲ on Neuronal Cell Injury. Guangzhou, China: Guangdong Pharmaceutical University.

[B99] Macdonald B. T. Tamai K. He X. (2009). Wnt/beta-catenin Signaling: Components, Mechanisms, and Diseases. Dev. Cel. 17 (1), 9–26. 10.1016/j.devcel.2009.06.016 PMC2861485 19619488

[B100] Magzoub M. (2020). Combating Proteins with Proteins: Engineering Cell-Penetrating Peptide Antagonists of Amyloid-β Aggregation and Associated Neurotoxicity. DNA Cel Biol 39 (6), 920–925. 10.1089/dna.2020.5604 32379486

[B101] Malhotra R. (2016). Understanding Migraine: Potential Role of Neurogenic Inflammation. Ann. Indian Acad. Neurol. 19 (2), 175–182. 10.4103/0972-2327.182302 27293326 PMC4888678

[B102] May B. H. Feng M. Zhou I. W. Chang S. Y. Lu S. C. Zhang A. L. (2016). Memory Impairment, Dementia, and Alzheimer's Disease in Classical and Contemporary Traditional Chinese Medicine. J. Altern. Complement. Med. 22 (9), 695–705. 10.1089/acm.2016.0070 27464225

[B103] Mcgeer P. L. Mcgeer E. Rogers J. Sibley J. (1990). Anti-inflammatory Drugs and Alzheimer Disease. Lancet 335 (8696), 1037. 10.1016/0140-6736(90)91101-f 1970087

[B104] Mengyong Z. Caijiao W. Husheng Z. Xianwu P. Jianmin F. (2008). Protective Effect of Polysaccharides from morinda Officinalis on Bone Loss in Ovariectomized Rats. Int. J. Biol. Macromol. 43 (3), 276–278. 10.1016/j.ijbiomac.2008.06.008 18638500

[B105] Mokry L. E. Ross S. Morris J. A. Manousaki D. Forgetta V. Richards J. B. (2016). Genetically Decreased Vitamin D and Risk of Alzheimer Disease. Neurology 87 (24), 2567–2574. 10.1212/WNL.0000000000003430 27856775 PMC5207000

[B106] Moon M. Song H. Hong H. J. Nam D. W. Cha M.-Y. Oh M. S. (2013). Vitamin D-Binding Protein Interacts with Aβ and Suppresses Aβ-Mediated Pathology. Cell Death Differ 20 (4), 630–638. 10.1038/cdd.2012.161 23257976 PMC3595489

[B107] Nanes M. S. Kallen C. B. (2014). Osteoporosis. Semin. Nucl. Med. 44 (6), 439–450. 10.1053/j.semnuclmed.2014.06.006 25362234

[B108] Ng A. Tam W. W. Zhang M. W. Ho C. S. Husain S. F. Mcintyre R. S. (2018). IL-1β, IL-6, TNF- α and CRP in Elderly Patients with Depression or Alzheimer's Disease: Systematic Review and Meta-Analysis. Sci. Rep. 8 (1), 12050. 10.1038/s41598-018-30487-6 30104698 PMC6089986

[B109] Park B. Song H. S. Kwon J. E. Cho S. M. Jang S. A. Kim M. Y. (2017). Effects of Salvia Miltiorrhiza Extract with Supplemental Liquefied Calcium on Osteoporosis in Calcium-Deficient Ovariectomized Mice. BMC Complement. Altern. Med. 17 (1), 545. 10.1186/s12906-017-2047-y 29262817 PMC5738837

[B110] Park E. Jin H. S. Cho D. Y. Kim J. Kim M. C. Choi C. W. (2014). The Effect of Lycii Radicis Cortex Extract on Bone Formation In Vitro and In Vivo. Molecules 19 (12), 19594–19609. 10.3390/molecules191219594 25432011 PMC6271141

[B111] Patel A. Kimura R. Fu W. Soudy R. Mactavish D. Westaway D. (2021). Genetic Depletion of Amylin/Calcitonin Receptors Improves Memory and Learning in Transgenic Alzheimer's Disease Mouse Models. Mol. Neurobiol. 58 (10), 5369–5382. 10.1007/s12035-021-02490-y 34312771 PMC8497456

[B112] Patel S. Homaei A. Raju A. B. Meher B. R. (2018). Estrogen: The Necessary Evil for Human Health, and Ways to Tame it. Biomed. Pharmacother. 102, 403–411. 10.1016/j.biopha.2018.03.078 29573619

[B113] Pei H. Ma L. Cao Y. Wang F. Li Z. Liu N. (2020). Traditional Chinese Medicine for Alzheimer's Disease and Other Cognitive Impairment: A Review. Am. J. Chin. Med. 48 (3), 487–511. 10.1142/S0192415X20500251 32329645

[B114] Peng X. M. Gao L. Huo S. X. Liu X. M. Yan M. (2015). The Mechanism of Memory Enhancement of Acteoside (Verbascoside) in the Senescent Mouse Model Induced by a Combination of D-Gal and AlCl3. Phytother. Res. 29 (8), 1137–1144. 10.1002/ptr.5358 25900087

[B115] Pinheiro L. Faustino C. (2019). Therapeutic Strategies Targeting Amyloid-β in Alzheimer's Disease. Curr. Alzheimer Res. 16 (5), 418–452. 10.2174/1567205016666190321163438 30907320

[B116] Prince M. Bryce R. Albanese E. Wimo A. Ribeiro W. Ferri C. P. (2013). The Global Prevalence of Dementia: a Systematic Review and Metaanalysis. Alzheimers Dement 9 (1), 63–e2. e2. 10.1016/j.jalz.2012.11.007 23305823

[B117] Püntener U. Booth S. G. Perry V. H. Teeling J. L. (2012). Long-term Impact of Systemic Bacterial Infection on the Cerebral Vasculature and Microglia. J. Neuroinflammation 9, 146. 10.1186/1742-2094-9-146 22738332 PMC3439352

[B118] Qi S. Zheng H. Chen C. Jiang H. (2019). Du-Zhong (Eucommia Ulmoides Oliv.) Cortex Extract Alleviates Lead Acetate-Induced Bone Loss in Rats. Biol. Trace Elem. Res. 187 (1), 172–180. 10.1007/s12011-018-1362-6 29740803

[B119] Ross R. D. Shah R. C. Leurgans S. Bottiglieri T. Wilson R. S. Sumner D. R. (2018). Circulating Dkk1 and TRAIL Are Associated with Cognitive Decline in Community-Dwelling, Older Adults with Cognitive Concerns. J. Gerontol. A. Biol. Sci. Med. Sci. 73 (12), 1688–1694. 10.1093/gerona/glx252 PMC6230213 29432613

[B120] Scali C. Caraci F. Gianfriddo M. Diodato E. Roncarati R. Pollio G. (2006). Inhibition of Wnt Signaling, Modulation of Tau Phosphorylation and Induction of Neuronal Cell Death by DKK1. Neurobiol. Dis. 24 (2), 254–265. 10.1016/j.nbd.2006.06.016 16919965

[B121] Singh Y. Gupta G. Shrivastava B. Dahiya R. Tiwari J. Ashwathanarayana M. (2017). Calcitonin Gene-Related Peptide (CGRP): A Novel Target for Alzheimer's Disease. CNS Neurosci. Ther. 23 (6), 457–461. 10.1111/cns.12696 28417590 PMC6492742

[B122] Song D. Cao Z. Liu Z. Tickner J. Qiu H. Wang C. (2018). Cistanche Deserticola Polysaccharide Attenuates Osteoclastogenesis and Bone Resorption via Inhibiting RANKL Signaling and Reactive Oxygen Species Production. J. Cel. Physiol. 233 (12), 9674–9684. 10.1002/jcp.26882 29968926

[B123] Sreenivasmurthy S. G. Liu J. Y. Song J. X. Yang C. B. Malampati S. Wang Z. Y. (2017). Neurogenic Traditional Chinese Medicine as a Promising Strategy for the Treatment of Alzheimer's Disease. Int. J. Mol. Sci. 18 (2). 10.3390/ijms18020272 PMC5343808 28134846

[B124] Steinhart Z. Angers S. (2018). Wnt Signaling in Development and Tissue Homeostasis. Development 145 (11). 10.1242/dev.146589 29884654

[B125] Stevenson J. C. (2006). HRT, Osteoporosis and Regulatory Authorities Quis Custodiet Ipsos Custodes? Hum. Reprod. 21 (7), 1668–1671. 10.1093/humrep/del043 16556675

[B126] Tamir S. Eizenberg M. Somjen D. Izrael S. Vaya J. (2001). Estrogen-like Activity of Glabrene and Other Constituents Isolated from Licorice Root. J. Steroid Biochem. Mol. Biol. 78 (3), 291–298. 10.1016/s0960-0760(01)00093-0 11595510

[B127] Tan Z. S. Seshadri S. Beiser A. Zhang Y. Felson D. Hannan M. T. (2005). Bone mineral Density and the Risk of Alzheimer Disease. Arch. Neurol. 62 (1), 107–111. 10.1001/archneur.62.1.107 15642856

[B128] van Dam P. A. Verhoeven Y. Trinh X. B. Wouters A. Lardon F. Prenen H. (2019). RANK/RANKL Signaling Inhibition May Improve the Effectiveness of Checkpoint Blockade in Cancer Treatment. Crit. Rev. Oncol. Hematol. 133, 85–91. 10.1016/j.critrevonc.2018.10.011 30661662

[B129] Wallin K. Solomon A. Kåreholt I. Tuomilehto J. Soininen H. Kivipelto M. (2012). Midlife Rheumatoid Arthritis Increases the Risk of Cognitive Impairment Two Decades Later: a Population-Based Study. J. Alzheimers Dis. 31 (3), 669–676. 10.3233/JAD-2012-111736 22647255

[B130] Walsh D. A. Mapp P. I. Kelly S. (2015). Calcitonin Gene-Related Peptide in the Joint: Contributions to Pain and Inflammation. Br. J. Clin. Pharmacol. 80 (5), 965–978. 10.1111/bcp.12669 25923821 PMC4631170

[B131] Wang D D. Wang H. Gu L. (2017). The Antidepressant and Cognitive Improvement Activities of the Traditional Chinese Herb Cistanche. Evid. Based Complement. Alternat Med. 2017, 3925903. 10.1155/2017/3925903 28744316 PMC5506466

[B132] Wang H. K. Hung C. M. Lin S. H. Tai Y. C. Lu K. Liliang P. C. (2014). Increased Risk of Hip Fractures in Patients with Dementia: a Nationwide Population-Based Study. BMC Neurol. 14, 175. 10.1186/s12883-014-0175-2 25213690 PMC4172891

[B133] Wang H. M. Wang L. Li N. (2004). Study the Influence of Morinda Officinalis How on the Differentiation From Marrow Stroma Cell to Osteoblast. Rehabil. Med. 14, 16–20.

[B134] Wang J J. Li Y. Huang W. H. Zeng X. C. Li X. H. Li J. (2017). The Protective Effect of Aucubin from Eucommia Ulmoides against Status Epilepticus by Inducing Autophagy and Inhibiting Necroptosis. Am. J. Chin. Med. 45 (3), 557–573. 10.1142/S0192415X17500331 28387136

[B135] Wang T T. Liu Q. Tjhioe W. Zhao J. Lu A. Zhang G. (2017). Therapeutic Potential and Outlook of Alternative Medicine for Osteoporosis. Curr. Drug Targets 18 (9), 1051–1068. 10.2174/1389450118666170321105425 28325144

[B136] Wang T. J. Zhang F. Richards J. B. Kestenbaum B. van Meurs J. B. Berry D. (2010). Common Genetic Determinants of Vitamin D Insufficiency: a Genome-wide Association Study. Lancet 376 (9736), 180–188. 10.1016/S0140-6736(10)60588-0 20541252 PMC3086761

[B137] Wang X. Z. (2012). Extraction, the Structure Modification of Atractylenolide and Protective Effect on PC12 Cells. [Master Thesis]. Guangzhou, China: Guangzhou University of Chinese Medicine.

[B138] Wang Z. Huang X. Zhao P. Zhao L. Wang Z. Y. (2018). Catalpol Inhibits Amyloid-β Generation through Promoting α-Cleavage of APP in Swedish Mutant APP Overexpressed N2a Cells. Front. Aging Neurosci. 10, 66. 10.3389/fnagi.2018.00066 29615891 PMC5867310

[B139] Weller I. Schatzker J. (2004). Hip Fractures and Alzheimer's Disease in Elderly Institutionalized Canadians. Ann. Epidemiol. 14 (5), 319–324. 10.1016/j.annepidem.2003.08.005 15177270

[B140] Weller J. Budson A. (2018). Current Understanding of Alzheimer's Disease Diagnosis and Treatment. F1000Res 7, 1. 10.12688/f1000research.14506.1 PMC6073093 30135715

[B141] Wojda S. J. Donahue S. W. (2018). Parathyroid Hormone for Bone Regeneration. J. Orthop. Res. 36 (10), 2586–2594. 10.1002/jor.24075 29926970

[B142] Wong W. (2020). Economic burden of Alzheimer Disease and Managed Care Considerations. Am. J. Manag. Care 26 (8 Suppl. l), S177–S183. 10.37765/ajmc.2020.88482 32840331

[B143] Xia Z. Wang F. Zhou S. Zhang R. Wang F. Huang J. H. (2017). Catalpol Protects Synaptic Proteins from Beta-Amyloid Induced Neuron Injury and Improves Cognitive Functions in Aged Rats. Oncotarget 8 (41), 69303–69315. 10.18632/oncotarget.17951 29050205 PMC5642480

[B144] Xiong W. Zhao L. (2016). Effect of Eucommia Ulmoides with Salt Water on Blood Biochemical Indexes in Senile Osteoporosis Rats. Lishizhen Med. Materia Med. Res.

[B145] Yaccoby S. Ling W. Zhan F. Walker R. Barlogie B. Shaughnessy J. D. (2007). Antibody-based Inhibition of DKK1 Suppresses Tumor-Induced Bone Resorption and Multiple Myeloma Growth In Vivo. Blood 109 (5), 2106–2111. 10.1182/blood-2006-09-047712 17068150 PMC1801040

[B146] Ye Z. X. Xiao L. Y. Pan J. Q. (2015). Effect of morinda Officinalis Extraction Learning and Memory Impairment in Mice. Guangzhou, China: Guangzhou Panyu sanatorium.

[B147] Yu M. S. Lai C. S. Ho Y. S. Zee S. Y. So K. F. Yuen W. H. (2007). Characterization of the Effects of Anti-aging Medicine Fructus Lycii on Beta-Amyloid Peptide Neurotoxicity. Int. J. Mol. Med. 20 (2), 261–268. 10.3892/ijmm.20.2.261 17611646

[B148] Yuen C. W. Murugaiyah V. Najimudin N. Azzam G. (2021). Danshen (Salvia Miltiorrhiza) Water Extract Shows Potential Neuroprotective Effects in *Caenorhabditis elegans* . J. Ethnopharmacol. 266, 113418. 10.1016/j.jep.2020.113418 32991971

[B149] Zhang H. Zheng Y. (2019). β Amyloid Hypothesis in Alzheimer's Disease:Pathogenesis,Prevention,and Management. Zhongguo Yi Xue Ke Xue Yuan Xue Bao 41 (5), 702–708. 10.3881/j.issn.1000-503X.10875 31699204

[B150] Zhang J. H. Xin H. L. Xu Y. M. Shen Y. He Y. Q. Hsien-Yeh H. (2018). Morinda Officinalis How. - A Comprehensive Review of Traditional Uses, Phytochemistry and Pharmacology. J. Ethnopharmacol. 213, 230–255. 10.1016/j.jep.2017.10.028 29126988

[B151] Zhang Nd N. D. Han T. Huang B. K. Rahman K. Jiang Y. P. Xu H. T. (2016). Traditional Chinese Medicine Formulas for the Treatment of Osteoporosis: Implication for Antiosteoporotic Drug Discovery. J. Ethnopharmacol. 189, 61–80. 10.1016/j.jep.2016.05.025 27180315

[B152] Zhang Q. Du X. Xu Y. Dang L. Xiang L. Zhang J. (2013). The Effects of Gouqi Extracts on Morris Maze Learning in the APP/PS1 Double Transgenic Mouse Model of Alzheimer's Disease. Exp. Ther. Med. 5 (5), 1528–1530. 10.3892/etm.2013.1006 23737913 PMC3671880

[B153] Zhang R. Pan Y. L. Hu S. J. Kong X. H. Juan W. Mei Q. B. (2014). Effects of Total Lignans from Eucommia Ulmoides Barks Prevent Bone Loss In Vivo and In Vitro. J. Ethnopharmacol. 155 (1), 104–112. 10.1016/j.jep.2014.04.031 24786573

[B154] Zhang Xz X. Z. Qian S. S. Zhang Y. J. Wang R. Q. (2016). Salvia Miltiorrhiza: A Source for Anti-alzheimer's Disease Drugs. Pharm. Biol. 54 (1), 18–24. 10.3109/13880209.2015.1027408 25857808

[B155] Zhao X. Wang Y. Nie Z. Han L. Zhong X. Yan X. (2020). Eucommia Ulmoides Leaf Extract Alters Gut Microbiota Composition, Enhances Short-Chain Fatty Acids Production, and Ameliorates Osteoporosis in the Senescence-Accelerated Mouse P6 (SAMP6) Model. Food Sci. Nutr. 8 (9), 4897–4906. 10.1002/fsn3.1779 32994951 PMC7500782

[B156] Zhao Y. Shen L. Ji H. F. (2012). Alzheimer's Disease and Risk of Hip Fracture: a Meta-Analysis Study. ScientificWorldJournal 2012, 872173. 10.1100/2012/872173 22629218 PMC3354728

[B157] Zhou X. L. Xu M. B. Jin T. Y. Rong P. Q. Zheng G. Q. Lin Y. (2019). Preclinical Evidence and Possible Mechanisms of Extracts or Compounds from Cistanches for Alzheimer's Disease. Aging Dis. 10 (5), 1075–1093. 10.14336/AD.2018.0815-1 31595204 PMC6764737

[B158] Zhu B. Zhang Q. L. Hua J. W. Cheng W. L. Qin L. P. (2018). The Traditional Uses, Phytochemistry, and Pharmacology of Atractylodes Macrocephala Koidz.: A Review. J. Ethnopharmacol 226, 143–167. 10.1016/j.jep.2018.08.023 30130541

[B159] Zwerina J. Redlich K. Polzer K. Joosten L. Krönke G. Distler J. (2007). TNF-induced Structural Joint Damage Is Mediated by IL-1. Proc. Natl. Acad. Sci. U S A. 104 (28), 11742–11747. 10.1073/pnas.0610812104 17609389 PMC1913858

